# Modelling the spread and mitigation of an emerging vector-borne pathogen: Citrus greening in the U.S.

**DOI:** 10.1371/journal.pcbi.1010156

**Published:** 2023-06-02

**Authors:** Viet-Anh Nguyen, David W. Bartels, Christopher A. Gilligan

**Affiliations:** 1 Department of Plant Sciences, University of Cambridge, Cambridge, United Kingdom; 2 United States Department of Agriculture, Animal and Plant Health Inspection Service, Plant Protection and Quarantine, Fort Collins, Colorado, United States of America; University of Notre Dame, UNITED STATES

## Abstract

Predictive models, based upon epidemiological principles and fitted to surveillance data, play an increasingly important role in shaping regulatory and operational policies for emerging outbreaks. Data for parameterising these strategically important models are often scarce when rapid actions are required to change the course of an epidemic invading a new region. We introduce and test a flexible epidemiological framework for landscape-scale disease management of an emerging vector-borne pathogen for use with endemic and invading vector populations. We use the framework to analyse and predict the spread of Huanglongbing disease or citrus greening in the U.S. We estimate epidemiological parameters using survey data from one region (Texas) and show how to transfer and test parameters to construct predictive spatio-temporal models for another region (California). The models are used to screen effective coordinated and reactive management strategies for different regions.

## Author summary

Citrus Greening or Huanglongbing (HLB) disease is caused by a bacterium invading the phloem of citrus trees. The disease is transmitted by the Asian citrus psyllid (ACP), a small invasive insect that feeds and lays eggs on the developing flush shoots in citrus. ACP was first found in the U.S. in Florida in 1998. Citrus greening was detected in Florida in 2005 and rapidly spread through commercial and residential citrus. Both the insect and disease have subsequently been detected and are spreading in the two other main citrus U.S. producing states, Texas and California. Both federal and state agencies in cooperation with the citrus industry and growers are working to slow or halt the spread of this devastating disease. Predictive models, as developed in this work, are increasingly important in shaping regulatory and operational policies for emerging outbreaks. Our model was develop using survey data from Texas and we show how to transfer and test the model parameters in both southern California and California’s central valley. The model can be used to screen coordinated and reactive management strategies, which help in the effort to slow the spread of the insect and disease.

## Introduction

A rapid response to emerging epidemics of crop disease is essential for successful management of an epidemic [1–3] just as for the pandemic of COVID-19 [4,5]. Effective management, however, requires knowledge of critical epidemiological parameters for transmission and dispersal of the pathogen [6–8]. Knowledge of epidemiological parameters is required for models to predict the current extent of infection, the likely future spread [7,9,10] and the effectiveness of potential intervention strategies [7,11–13]. Epidemiologically important parameters such as transmission rates and dispersal kernels are frequently unknown when an epidemic invades a new region. There are two options to obtain values for the epidemiological parameters: wait until there are adequate surveillance data in the newly invaded region from which to estimate parameters, or transfer parameters derived for another region and incorporate them into models that allow for different host distributions and environmental variables in the newly invaded region. Here we introduce and test an epidemiological modelling framework for an emerging epidemic of Huanglongbing (HLB) disease, one of the most serious threats to citrus production world-wide [14,15]. We parameterise and validate the model using training and test data in one region (Lower Rio Grande Valley in Texas). We then assess the adaptability and validation of the model in new regions with different climatic conditions, where the insect vector is endemic (southern California) or invading (Central Valley, California).

Huanglongbing disease also known as citrus greening causes severe chlorosis of foliage, dieback, loss of yield, discolouration and ill-flavour of fruit, and death of citrus trees [14]. The disease is associated with three bacterial strains of which *Candidatus* Liberibacter asiaticus (Las) is the prevalent type in the Western Hemisphere and the only strain that has been reported as established within the U.S. The disease is reported to have caused a 74% drop by 2018 in citrus production in Florida since the first detection of the pathogen in 2005 (USDA/NASS; 2022). The pathogen has spread rapidly in backyard and commercial trees in Texas [16] and has been introduced and become established in backyard trees in southern California [17,18]. There is a consequent risk of invasion into the major citrus production area with the Central Valley in California [19], where the insect vector, the Asian citrus psyllid (ACP), *Diaphorina citri* Kuwayama, is currently invading. The pathogen also continues to pose a major threat to citrus production in Brazil [20]. The invasion of another vector, the African citrus psyllid, *Trioza erytreae* (Del Guercio), which primarily transmits the Africanus strain of Liberibacter (Laf), in Portugal and Spain constitutes a threat of introduction of the disease into European countries [21]. The disease is also widespread in South East Asia [22] and the Las bacterium has recently been reported in Kenya [23].

**Fig 1:**
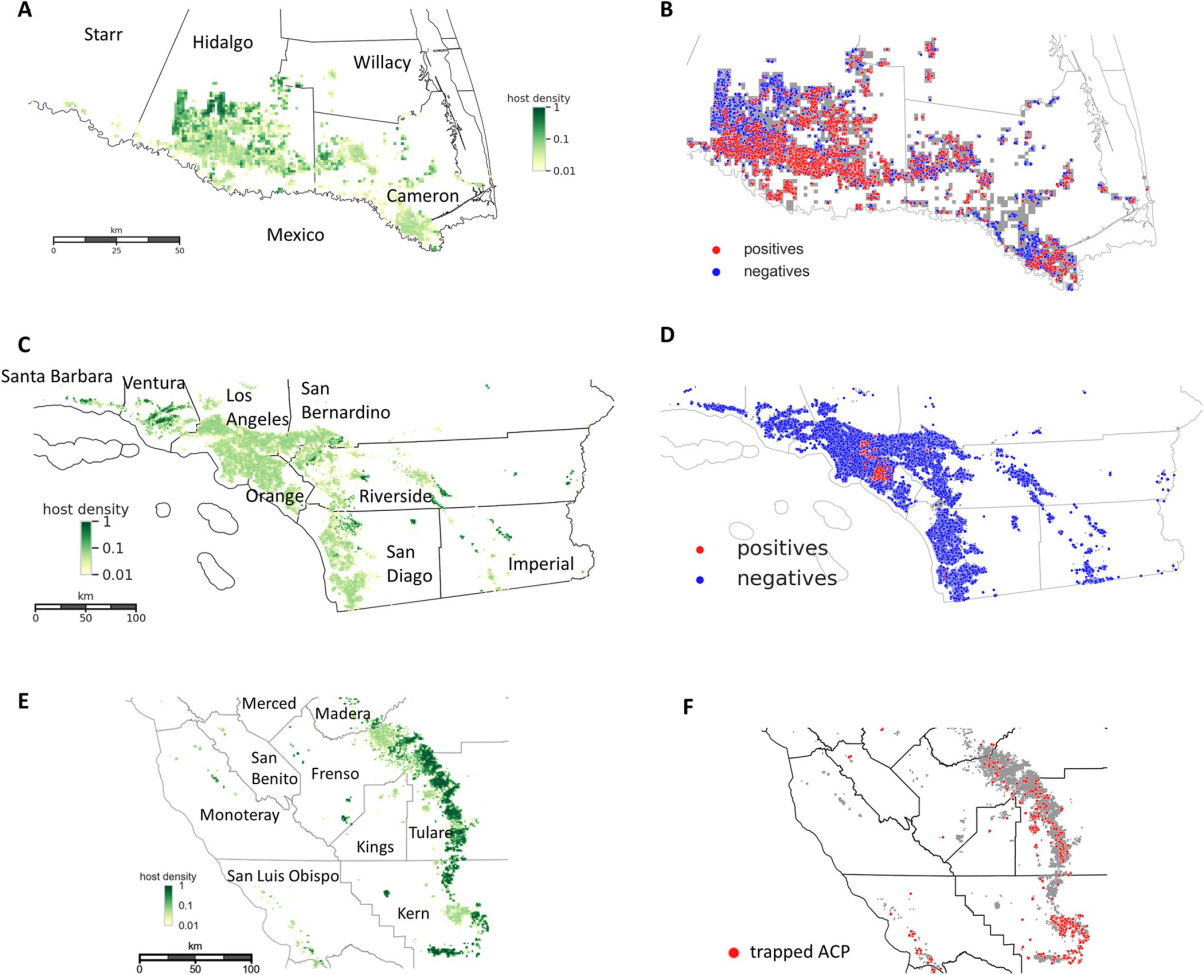
The citrus landscapes for three regions in Texas and California and the survey data for emerging HLB and ACP epidemics in the regions. (A.C,E) Gridded approximation of the citrus distribution in the (A) Lower Rio Grande Valley, Texas, (C) southern California, and (E) the Central Valley. The regions comprise large commercial citrus groves (high host density) interspersed with dooryard trees in residential areas (low host density). (B,D,F) Geo-coded diagnostic plant samples collected as part of the (B) Texas HLB state-wide survey between December 2011 and October 2018, (D) California state-wide survey between June 2015 and June 2019. We classed samples with Ct value less than 36 (out of 40 qPCR cycles) as positives. (F) Geo-coded ACP samples recorded by California Department of Food and Agriculture independently from the state-wide HLB survey. The samples were found in sticky yellow traps set up near trees in the Central Valley from 2012 to 2017 inclusively. Basemap shapefile for cartographic boundaries reproduced from U.S. Census Bureau under open data use.

The pathogen is transmitted by human mediated movement of infected planting material as well as the insect vector [14]. There are currently no genetically resistant hosts. Options for control include pesticide application to kill the vector, removal of infected and surrounding trees and quarantine to prevent movement of infected planting material and citrus fruit [24]. Given the widespread distribution and continued spread of the pathogen and the psyllid vectors, there is an urgent need for a flexible parameterised epidemiological model that can be used to predict spread at landscape scales and to inform surveillance and management options. Here we adopt a classical susceptible-exposed-infected (SEI) compartmental modelling framework for HLB [1,8]. We couple this with a model for the dynamics of ACP infection and an observational model for vector trapping data and disease surveillance data. The models take account of the heterogeneous distribution of the citrus host in the landscape, encompassing plantations and backyard trees [25]. The models are parameterised using Bayesian methods and extensive surveillance data that comprises successive snapshots of disease for the citrus growing region in the lower Rio Grande Valley in Texas (Fig 1A and 1D).

The Bayesian approach allows for commonly encountered problems in the estimation of parameters for emerging epidemics. These include incomplete spatial coverage of surveys that require inferences about chains of unobserved infections between recorded snapshots of disease in the landscape and allowance for cryptic infection from asymptomatic or otherwise undetected hosts [7,25,26]. The parameter estimation also takes account of the confounding effects of pesticide application by some growers to manage the vector, which affects the observed dynamics of pathogen spread [8]. Our initial objective is to test and validate the model using the Texas data. Specifically, we use the model to quantify the infection pressures from primary and secondary infection sources in order to analyse the roles of different transmission mechanisms in epidemic spread. We also use the model to analyse the effectiveness of ACP control strategies retrospectively for the epidemic in Texas.

The HLB epidemic in southern California is at an earlier stage compared with the outbreak in Texas. The ACP vector was first detected in southern California in 2008 [27] and is now endemic. HLB was first detected in 2012 in Hacienda Heights and then in 2015 in the San Gabriel areas of greater Los Angeles with subsequent clusters of infection in Los Angeles, Orange, Riverside and San Bernardino counties. An extensive surveillance programme linked with compulsory treatment and removal of infected trees together with imposition of quarantines over movements of planting material and fruit around infected sites has restricted but not prevented the spread of the disease. The reservoir of the pathogen in southern California poses a threat to the principal citrus production area for the state in the Central Valley, where the ACP vector is known to be invading [17]. The intensive management of the disease in southern California along with absence in the Central Valley mean there were insufficient data to re-estimate parameters for the epidemiological models under Californian conditions. Accordingly, we therefore test the adaptability and validation of the model in new regions with different climatic conditions. We use the model to predict rates of pathogen and vector spread and screen some options for management. The potential flexibility of the combined modelling and parameter estimation are discussed.

## Methods

### Data

**Fig 2:**
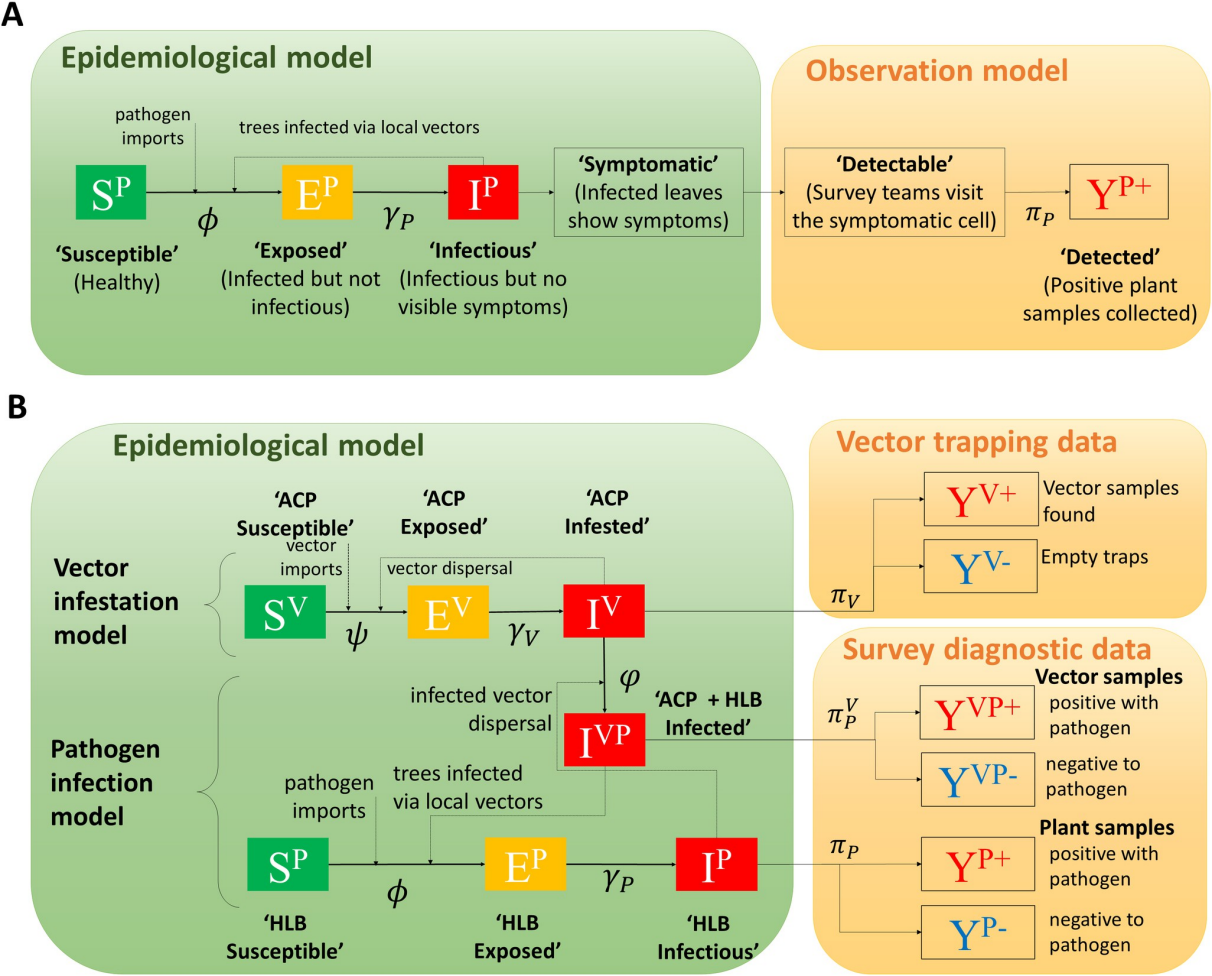
Epidemiological models for ACP and HLB spread. (A) The stochastic compartment model for HLB epidemiological dynamics in a Texas citrus grid cell and the observation model that matches infection status to survey diagnostic data. An infectious cell can infect other susceptible cells via the movement of local vectors, which were known to have established over the whole region before the emergence of HLB. *ϕ*, *γ*_*P*_, *π*_*P*_ denote the transition rates and detection probability and are described in Table 1 and derived in the Methods section. (B) The joint epidemiological model for ACP and HLB dynamics in California where ACP is invading and not yet endemic. The model extends the Texas model and introduces a new epidemic category ‘ACP + HLB infected’ that connects the dynamics of ACP infestation to HLB infection in a grid cell. The transition rates *ϕ*, *ψ*, *φ*, *γ*_*V*_, *γ*_*P*_ and detection probabilities $\pi_{V},\pi_{P},\pi_{P}^{V}$ are described in Table 1 and derived in the Methods section.

We obtained data from surveys for early HLB detections in both plant and psyllid samples in Texas and California and detection of ACP invasion in California. The data were collected by the U.S Department of Agriculture (USDA) and the California Department of Food and Agriculture (CDFA). Data collection surveys were part of an intensive area-wide management regulatory program for HLB over multiple U.S. states and localized management of ACP in California. The survey teams collected leaves with HLB-like symptoms and psyllids based on a risk-based survey model [28]. Samples were tested for Las bacteria in certified laboratories using the approved USDA diagnostic protocol utilizing qPCR technology [29]. For confirmatory diagnostic tests of plant tissue samples, a Ct value less than 36 (over 40 cycles) classified the sample as HLB positive. A Ct threshold of 38 was used for samples of vectors. The lower threshold for leaf tissue samples was an internal policy decision by USDA for added confidence in the diagnostic results because USDA regulates on positive plant tissue samples, but not on ACP samples positive for Las bacteria. We used the HLB leaf tissue survey data in Texas (Fig 1A and 1B) to estimate parameters and validate an HLB epidemiological spread model (Fig 2A). We also used an additional survey of psyllid samples for HLB-infected ACP in Texas to estimate parameters for vector spread (S1B Fig). Maps of citrus distribution (Fig 1C and 1D), together with HLB survey and ACP trapping data for southern California (Fig 1D) and Central Valley (Fig 1F) were used to initiate simulated epidemics for model inference on spread and control strategies and for further validation. All data were geo-mapped and aggregated by visiting dates on rasterised 1 km x 1 km grid cells to obtain spatiotemporal datasets for presence or absence of HLB for plant and vector samples in Texas (Figs 1B and S1B)), for presence or absence of HLB plant samples in southern California (Fig 1D) and ACP presence in the Central Valley (Fig 1F). For further details of the datasets used for parameter estimation, see S1 Text.

**Table 1:** Principal variables and parameters used in the models, together with estimated values for parameters derived from data augmented Markov chain Monte Carlo inference (see text for details).

Symbol	Description		Estimated Values
**Parameters estimated using DA-MCMC on Texas data**			
	**HLB Epidemiological Model**		
*ϵ*	Rate of HLB primary infection by trade and other human-mediated activities within the region of interest		$1.90_{1.1}^{2.7}\times 10^{-5}$ per day
*ε*_*B*_	Rate of HLB primary infection from cross-border psyllid transmission for cells along the Mexico border		$4.65_{0.31}^{13.15}\times 10^{-4}$ per day
*ε*_*W*_	Rate of HLB primary infection from wider psyllid transmission from outside the region of interest		$2.7_{0.1}^{14.2}\times 10^{-5}$ per day
*α*	Spatial dispersal scale for ACP movements		$1.96_{1.61}^{2.48}$ km
*β*_*P*_	Rate of HLB secondary infection		$0.33_{0.27}^{0.41}$ per day
*μ*	Ratio of feeding vs. moving time of vectors		$5.14_{0.53}^{26.37}\times 10^{-3}$
*η*	Efficiency of the annual coordinated ACP spraying program in commercial orchards		$79.6_{75.1}^{83.0}$ (%)
*σ* ^−1^	Duration for an HLB infectious cell to get detected by state-wide visual inspection survey		$469.0_{420.0}^{546.0}$ days
	**Observation model parameters**		
*π*_*p*_	Probability of detection of HLB from an infectious site		$46.3_{44.6}^{48.1}$ (%)
$\pi_{p}^{V}$	Probability of detection of ACP that carries HLB pathogen from an infectious site		$0.57_{0.07}^{0.97}$ (%)
*π*_*V*_	Probability of finding trapped ACP from an infested site	-^1^	
	**Additional parameters for combined HLB-ACP model**		
*ε*_*V*_	Rate of ACP primary infestation	$2.7_{0.1}^{14.2}\times 10^{-5}$ per day	
*β*_*V*_	Rate of ACP secondary infestation		$0.78_{0.61}^{1.03}$ per day
*ξ*	Rate of vectors picking up Las from local infectious trees within a citrus grid cell		$2.03_{2.19}^{2.36}\times 10^{-3}$ per day
**Empirical previously estimated parameters** ^**2**^			
$a_{p}^{-1}$	HLB Exposed to Infectious period		15 months
*a*_*V*_	ACP Exposed to Infestation period		15 days
**Derived parameters**			
*ϕ*_*i*_	Rate of HLB exposure or the overall HLB infection pressure for a cell *i* with endemic ACP		
*ψ*_*i*_	Rate of ACP exposure or the overall ACP infestation pressure		
*φ*_*i*_	Rate of HLB exposure for a cell *i* with epidemic ACP		
**Modelling variables**			
	**Grid cells**		
*i*, *j*, *j*′	Citrus grid cell indices		
*b*_*i*_	Binary indicator whether cell *i* is adjacent to the Mexico border		
*h*_*i*_	Citrus density in cell *i*		
*r*_*ij*_	Spatial distance between cells *i* and *j*		
	State variables		
$S_{i}^{P}(t)$	HLB susceptibility status of cell *i* at time *t*		
$E_{i}^{P}(t)$	HLB exposed status of cell *i* at time *t*		
$I_{j}^{P}(t)$	HLB infectious status of cell *j* at time *t*		
$S_{i}^{V}(t)$	ACP susceptibility status of cell *i* at time *t*		
$E_{i}^{V}(t)$	ACP exposed status of cell *i* at time *t*		
$I_{j}^{V}(t)$	ACP infestation status of cell *j* at time *t*		
$I_{j}^{VP}(t)$	HLB infectious status of ACP infested cell *j* at time *t*		
*κ*_*j*_	Vector density coefficient, which is the product of both vector control and weather suitability, for cell *j*		
$\kappa_{i}^{C}$	Vector control coefficient for cell *i*		
$\kappa_{i}^{W}$	Coefficient of weather suitability for vector development for cell *i*		
$f_{i}^{C}$	Proportion of commercial citrus trees in cell *i*		
*w*_*id*_	Temperature on day *d* in cell *i*		

^1^Parameter included in model for generality: not required in current fitting.

^2^Previously estimated by Parry et al. [8]: see S1 Text for further information.

### Epidemiological models

We used spatially explicit, continuous-time, stochastic compartment models for HLB and ACP dynamics and spread through rasterised landscapes comprising 1 km x 1 km grid cells. We treat the grid cells as the units of interest (i.e. in determining whether or not a grid cell is infected). We allow for the dynamics of infection spread within cells, i.e. transitions from Exposed to Infections to Symptomatic at the grid cell level but we do not explicitly model infections spread from one tree to another within grid cells. We also distinguish between two cases where the vector is endemic (Fig 2A: Texas and southern California) and where the vector is also spreading (Fig 2B: Central Valley, California). In addition, we also model the spread of HLB infected ACP through an endemic ACP population (S1B Fig). We link the HLB (Fig 2A) and HLB-ACP (Fig 2B) to observation models for detection of infected plants and infected vectors for the purposes of parameter estimation. The principal variables and parameters used in the models are summarised in Table 1. The simulation models and the algorithms for parameter estimation were coded in open access Python and are available in GitLab (see SI).

#### Pathogen spread by endemic insect vector

When the vector is endemic, as in Texas and southern California, we consider four categories for the status of HLB infection in each citrus grid cell: ‘HLB susceptible’, ‘HLB exposed’, ‘HLB infectious’, and ‘HLB detected’ (Fig 2A). A cell is susceptible if all the trees in the cell are healthy or free from the Las bacterium. An exposed cell contains infected trees but is not yet infectious to trees in other cells. An infectious cell can transmit Las bacteria to other cells via the movement of the psyllid (ACP). Finally, a cell becomes detected as a survey team collects a positive HLB sample confirmed by a qPCR diagnostic test. The transition of a cell from being infectious to detected requires two steps: first, infectious trees must show symptoms, and second, the site must be visited by a survey team in searching for the symptomatic trees.

#### Pathogen spread by invading insect vector

The model for HLB spread (Fig 2A) was adapted to account for the fact that the underlying ACP population in the Central Valley is still spreading and has not fully invaded the region. Grid cells were additionally classified (Fig 2B) as: ‘ACP susceptible’ (free from vectors), ‘ACP exposed’ (first vectors arrived in the cell but have not reproduced sufficiently to invade other cells), and ‘ACP infested’ (vectors settled in the cell and started invading nearby cells). We also introduced a new epidemic category ‘ACP + HLB infected’ for grid cells occupied by HLB-infected vectors (Fig 2B).

#### Modelling HLB exposure

An ‘HLB susceptible’ cell, *i*, is exposed to infection as the first tree in the cell gets infected. The exposure can happen either by primary or secondary transmission. Primary transmission originates from either the introduction of infected trees and products by trade and other human-mediated movements or from infected vectors arriving from external environments, including across the Mexico border. The border was marked by 1km^2^ cells that contain the border line. We introduce a parameter, *ϵ* to represent HLB importation rate by human activities and two parameters, *ε*_*B*_ for cross- border vector transmission and *ε*_*W*_ for vector transmission from further outside the region of interest.

Secondary transmission from an HLB infectious to a susceptible cell happens by the flux of vectors moving between the two cells. We denoted *β*_*P*_ as the rate for secondary infection and used an isotropic exponential kernel, *K*_*α*_(*r*)∝*e*^−*r*/*α*^ where *α* represents the dispersal scale, to depict the dependence of movement rate on the spatial distance, *r*, between the cells. The number of vectors moving towards a cell *i* from cell *j* depends on the distance between the cells and the availability of new flushes (foliar growth attractive for psyllid feeding) in the destination cell *i*. We established the overall strength of spatial coupling between grid cells by considering a mechanistic model that addresses vector movement and feeding. The model constructs the equilibrium abundance of psyllids moving to and feeding in a citrus grid cell. In doing so, it considers the rates of dispersal and also the birth and death rates of vectors (see S1 Text for a detailed derivation of the flux model). As vector dynamics occur at faster rates than HLB epidemiological dynamics, the vector counts quickly converge to equilibrium values that we used in calculating the HLB exposure rate (*ϕ*_*i*_(*t*): see also Fig 2A and 2B) from primary and secondary sources as follows:

$$\phi_{i}(t)=\varepsilon +[(1-b_{i})\varepsilon_{W}+b_{i}\varepsilon_{B}]h_{i}+\beta_{P}h_{i}\sum_{j}I_{j}^{V}(t)I_{j}^{P}(t)\frac{\kappa_{j}h_{j}K_{\alpha }(r_{ij})}{\mu +\Sigma_{j^{\prime }}\kappa_{j^{\prime }}h_{j^{\prime }}K_{\alpha }(r_{j}{}_{j^{\prime }})}$$ (1)

Secondary infection is encompassed in the final term in Eq (1) and accounts for the net exposure rate of a susceptible cell *i* from infectious cells *j*, allowing for citrus density, *h*_*i*_, and a vector density weight, $\kappa_{i}=\kappa_{i}^{C}\kappa_{i}^{W}$, which incorporated the effect of vector control, $\kappa_{i}^{C}$, and weather suitability to vector development $\kappa_{i}^{W}$. The superscripts *V* and *P* indicate infected vectors and plants, respectively and *j*′ is a general cell index that points to all cells in the host raster including *i* and *j*. The parameter *μ* is the ratio of time spent feeding compared with moving by the vector. See S1 Text for the derivation of Eq 1.

As commercial growers in Texas applied sprays in November and early February in a coordinated manner, they were able to reduce the density of vectors in commercial orchards. We used $\kappa_{i}^{C}$ to represent the relative weight of vector capacity in plantations compared with residential trees. We assumed that the vector density in cell *i* decreased as the proportion of commercial trees in the cell, $f_{i}^{C}$, increased. The parameter *η* denotes the efficiency of vector control measures applied to a cell:

$$\kappa_{i}^{C}=1-\eta f_{i}^{C}.$$

Daily temperatures and other weather variables affect vector density by leveraging or slowing down the development of eggs, nymphs, and adult vectors. We used $\kappa_{i}^{W}$ to account for the variation of vector density at different locations due to their corresponding weather patterns. We used the modified Logan function *r*(·) provided by Liu and Tsai [30] to calculate the vector development rate in a day, given the day’s temperatures, *r*_*id*_ = *r*(*w*_*id*_). The function addresses temperatures in the range of 10 to 33°C and assumes that no vector growth occurs beyond this range. We computed the expected weather-driven vector capacity coefficient, $\kappa_{i}^{W}$, by averaging over the development rates for the whole year:

$$\kappa_{i}^{W}=\sum_{d=1}^{365}r_{id}/365.$$

The unknown parameters *ϵ*, *ε*_*B*_, *ε*_*W*_, *α*, *β*_*P*_, *μ*, *η* required to model HLB exposure were estimated from the plant diagnostic data of the Texas HLB survey using a data augmented Markov chain Monte Carlo (DA-MCMC) algorithm under a Bayesian inference framework. We used uninformative prior distributions for all parameters.

#### Modelling ACP exposure

An ‘ACP susceptible’ cell *i* is exposed to an infestation when the first few vectors arrive in the cell either from nearby sites or are transported from external environments. We refer to these mechanisms as secondary and primary infestation and used parameters *β*_*V*_ and *ε*_*V*_, respectively, to represent the rates of infestation. We calculated the force of ACP exposure (*ψ*_*i*_(*t*): See also Fig 2B) to a susceptible cell *i* analogously to the pressure of HLB exposure (Eq 1) as follows (see S1 Text for a detailed derivation):

$$\psi_{i}(t)=\varepsilon_{V}h_{i}+\beta_{V}h_{i}\sum_{j}I_{j}^{V}(t)\frac{\kappa_{j}h_{j}K_{\alpha }(r_{ij})}{\mu +\Sigma_{j^{\prime }}\kappa_{j^{\prime }}h_{j^{\prime }}K_{\alpha }(r_{j}{}_{j^{\prime }})}$$ (2)

Since the ACP spread model was used for the Central Valley only, we discarded the extra risk of primary infection along the Mexico border. Lack of sufficient data meant we could not estimate the unknown parameters *ε*_*V*_, *β*_*V*_ directly from California ACP trapping data. We assumed, therefore, that *ε*_*V*_≈*ε*_*W*_ and estimated *β*_*V*_ from the Texas HLB survey data using the ‘ACP Infested’-to-‘ACP + HLB Infected’ model described below.

#### Modelling the transition from ‘ACP Infested’ to ‘ACP + HLB Infected’

An ‘ACP infested’ cell transitions to ‘ACP + HLB Infected’ cell (Fig 2B) when vectors in cell *i* acquire Las bacteria either by feeding on infectious trees inside the cell or by migrating from a nearby infectious cell. The former is also known as the bulking up of infected vectors inside the cell and is driven by an unknown parameter *ξ*. The rate at which infected vectors migrate from an ‘ACP + HLB Infected’ cell to an ‘ACP Infested’ site is equivalent to the rate *β*_*V*_ at which vectors from an ‘ACP Infested’ cell arrive in an ‘ACP Susceptible’ cell under the assumption that Las-carrying vectors behave similarly to uninfected vectors. Using the same dynamic model of vector movement and feeding as before, we can calculate the transition force as follows:

$$\varphi_{i}(t)=S_{i}^{P}(t)h_{i}\left(\xi \frac{I_{i}^{V}(t)E_{i}^{P}(t)}{\mu +\sum_{i^{\prime }\neq i}\kappa_{i^{\prime }}h_{i^{\prime }}K_{\alpha }(r_{ii^{\prime }})}+\beta_{V}\sum_{j\neq i}I_{j}^{V}(t)I_{j}^{P}(t)\frac{\kappa_{j}h_{j}K_{\alpha }(r_{ij})}{\mu +\sum_{j^{\prime }\neq j}\kappa_{j^{\prime }}h_{j^{\prime }}K_{\alpha }(r_{jj^{\prime }})}\right)$$ (3)

The superscripts *V* and *P* indicate the epidemic compartments for the vector and pathogen respectively. The unknown parameters *ξ*, *β*_*V*_ were estimated using the vector diagnostic data from the Texas HLB survey. Since we carried out parameter estimation under a Bayesian inference framework, we can use the previously acquired posterior estimates for *α*, *μ*, *η* to complement the sparsity of vector diagnostic data.

#### Latent period parameters

The time it takes for a cell to transition from ‘HLB Exposed’ to ‘HLB Infectious’ is known as the HLB latent period. It starts when a tree gets exposed to HLB infection and ends as a significant number of trees in the cell became infectious so that the number of Las-carrying vectors that move away from the cell is large enough to cause infection in another cell. We followed Parry et al. [8] to use a seasonally-forced model for the rate of infectiousness onset,

$$\begin{array}{c} \gamma (t)=2a_{P}{sin}^{2}\frac{\pi t}{365}, \end{array}$$ (4)

where *a*_*P*_ denotes the average rate at which a cell moves from ‘HLB Exposed’ to ‘HLB Infectious’. We set the parameter to empirical estimates from in-orchard and in-nursery observations for trees more than ten years of age, which reported an average latent period of 15 months, i.e. *a*_*P*_ = 0.8. We assumed that the specific latent period for each cell follows an exponential distribution with rate *γ*(*t*). As such, the latent periods vary among cells and include very short durations due to the exponential form of the model.

Analogously, the ACP latent period indicates the time it takes for a cell to transition from ‘ACP Exposed’ to ‘ACP Infested’. We used the same seasonally forced model as for the HLB latent period model, with *a*_*P*_ replaced by *a*_*V*_ = 0.042. The rate is equivalent to an expected ACP latent period of 15 days, which covers the duration for nymphs to develop into adult psyllids.

### Parameter estimation

#### MCMC details

We adopted a Bayesian approach to estimate parameter values for the epidemiological parameters from the survey data. We treated the unknown timing of epidemiological transitions as random variables and used a data augmented MCMC algorithm [31,32] to infer the timings of the unobserved transitions. The Metropolis-Hasting method [33] was used to construct samplers for both parameters and unobserved epidemic transitions. We used simple log-normal proposal distributions for *ϵ*, *ε*_*W*_, *ε*_*B*_, *α*, *μ*, *η*, *ξ*, *σ*, *π*, and Gibbs samplers for *β*_*P*_, *β*_*V*_. We used the randomized construction of Markov trajectory [34] and exact inference algorithms for hidden Markov models [35] to improve samplers of epidemic transitions. Further details of the algorithms are given in the SI.

The overall likelihood of a set of parameter values comprises the model likelihood and the data likelihood (Fig 2B). The model likelihoods (Eqs (5) and (8) in the SI) can be naturally derived from the stochastic construction of the HLB and ACP epidemiological models described above. We developed data likelihoods (Eqs (6, 7 and 9) in the SI) using two parameters of the data collection process: *π* represents the probability that a positive sample is collected from an infectious cell, and *σ* indicates the expected duration from becoming infectious to getting detected. Both parameters, together with parameters of epidemiological models, were estimated from Texas HLB survey data.

### Model validation

We estimated the epidemiological parameters for spread of HLB (*ϵ*, *ε*_*W*_, *ε*_*B*_, *α*, *β*_*P*_, *μ*, *η*, *σ*, *π*) using the Texas HLB survey data collected between December 2011 and August 2016 as training data. We validated the model using data collected between September 2016 and October 2018 as the testing data for Texas. The application of the HLB spread model, parameterised for Texas to southern California was validated using surveillance data collected for HLB collected in southern California from June 2015 to June 2019. We compared model simulations against testing data in terms of both temporal progression and spatial autocorrelation metrics. We used Moran’s I [36,37]. See the S1 Text for further details of the validation processes.

The application of the ACP spread model, parameterised using Texas plant and vector diagnostic data and adjusted for temperature conditions, to the Central Valley, was validated against ACP trapping data for the Central Valley in 2015 and 2016. We used temporal prevalence as the evaluation metric and compared the model predictions with the observed trapping data.

### Model variants

To analyse the sensitivity of different model components to prediction performance, we considered four variants to the full model described above. Each model variant differed from the full model by one component of the secondary transmission model: (1) no normalisation, in which the normalisation term for vector fluxes is assumed to be the same for all cells and absorbed into the secondary infection rate; (2) no control effect, in which we ignored the occurrence of the annual coordinated spraying program; (3) no border effect, in which we did not distinguish between sites near to and far from the Mexico border and used the same primary infection rate for infected vectors from external environments; (4) power-law kernel, in which the exponential dispersal function is replaced with a power-law function. Model variants were fitted using the Texas HLB survey data up to August 2016 and validated with data up to August 2017 (S2 and S3 Figs).

### Predictions and allowance for control

We made prospective predictions more than two years beyond the final observation time for each region. Not all sites already infected with HLB are observable at the time of forecast. The observed survey data were used to infer the locations of cells that had been exposed to and infectious with HLB at that time. We used an MCMC-based simulator analogous to the data-augmented MCMC algorithms used for parameter estimation to sample epidemic transition times that agreed well with observed data.

Model simulations were also used to analyse the efficiencies of selected control measures in each region. These included an annual coordinated spraying program to manage ACP in Texas, reactive removal of infected trees and HLB quarantine in southern California, and reactive vector spraying upon ACP detection from sticky traps in the Central Valley. The efficiency of ACP control strategies in Texas was assessed by comparing results for *η* = 100%, 50%, 20% of the estimated (default) value for the spread of HLB between 2011 and 2019. For southern California, we simulated epidemic trajectories with a quarantine radius of 1, 2, 3, …, 14 km. The potential of the reactive ACP eradication program in the Central Valley was assessed by varying the treatment efficiency between 0% and 100% and the eradication radius from 0.1 to 2.0 km. In all cases 1000 simulations were run for each scenario and credible intervals 50%, 75% and 95% for disease trajectories were computed. (See S1 Text for further details of simulations.)

## Results

### Parameterisation and validation of the model for the HLB epidemic in the Lower Rio Grande Valley, Texas

**Fig 3:**
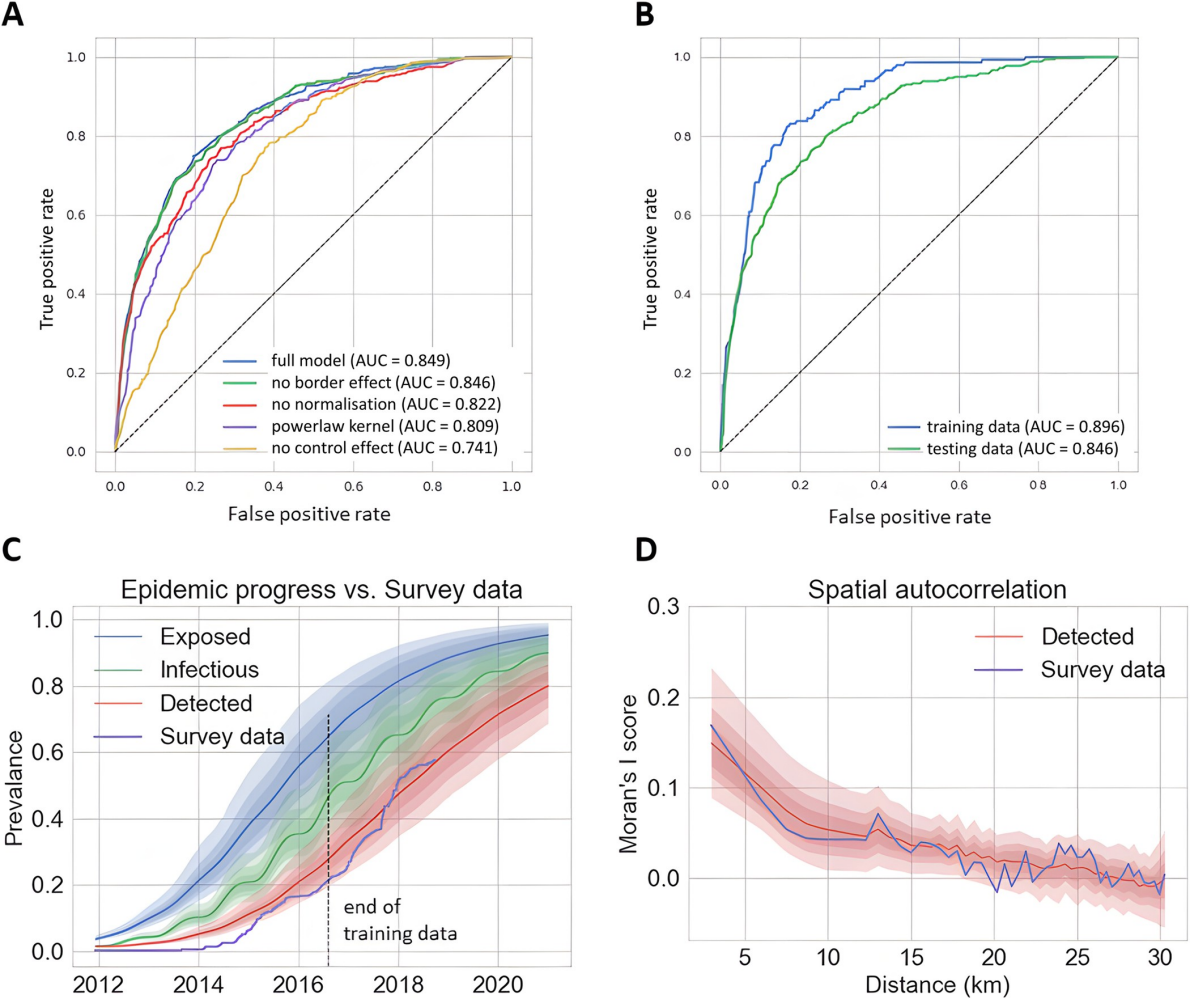
Model selection and goodness-of-fit of the full HLB spread in the Lower Rio Grande Valley, Texas. (A) Performance of the full model and four model variants in predicting the outcome of survey trials assessed by receiver operating characteristic (ROC) curves. The area under the curve (AUC) of the ROC curve measures the predictive capability of calibrated models when used as binary classifiers to separate positive from negative (1km x 1km) sites. Models were fitted to a randomly sampled portion of survey data up to August 2016, and tested using the remaining portion and data up to August 2017. (B) Performance of the full model in predicting the outcome of survey trials within and beyond the temporal scope of the Texas dataset used for parameter estimation in Table 1. The model was fitted to a randomly sampled portion (80%) of the training data (collected between December 2011 to August 2016) and tested using the remaining part (20%) of the training data and the testing data (collected between September 2016 and October 2018) for the ROC analysis. (C) Temporal progression of the prevalence of three infection categories (Exposed, Infectious, and Detected) for the full model compared with the surveillance data. Besides the medians of 1000 simulation realizations (solid lines), we also show 50%, 75%, and 95% credible intervals (shades of decreasing intensities). The vertical dotted line separates the training dataset (used for parameter estimation) from the test dataset. (D) Spatial autocorrelation scores using Moran’s I of the ‘detected’ categories of the model with the survey data at the end of August 2017: medians of 1000 simulation realizations (red line) with 50%, 75%, and 95% credible intervals (shades of decreasing intensities).

Preliminary analyses were carried out to compare the performance of the full HLB epidemiological model, as specified in Fig 2A, with four variants comprising: (i) no normalisation for vector flux; (ii) no allowance for vector control in commercial plantations; (iii) no border effect on primary infection rates; (iv) use of a power-law instead of an exponential dispersal kernel. The epidemiological parameters were estimated for each model from the training dataset using the DA-MCMC algorithm within a Bayesian inference framework to approximate the joint posterior distributions of the parameters. The goodness-of-fit to the test data was assessed by inspection of the temporal progression of the ‘Detected’ cells with the observed surveillance data (S2 Fig) for each model. Spatial autocorrelation scores of the ‘detected’ categories were compared with the survey data at the end of August 2017, using Moran’s I (S3 Fig). We also used the receiver operating characteristics (ROC) curve analysis to compare and evaluate the models’ predictive performance (Fig 3A).

The model variants, based upon 500 simulations, captured the temporal progress of disease broadly similarly (S2 Fig) but differed in the evaluation of the spatial metrics (S3 Fig) and the ROC curves. The variants with no normalisation, no control effect and a power-law kernel each performed less well than the full model and the variant with no border effect (S3 Fig). The ROC curves (Fig 3A) indicated superior performance for the full model and the variant with no border effect over the other three variants, with the full model performing marginally better that the ‘no-border’ variant (Fig 3A).

The full model was selected for future work and the parameters re-estimated with a slightly adjusted citrus landscape to accommodate for updated information of residential sites and with 1000 simulations. We used the data-augmented Markov chain Monte Carlo (DA-MCMC) algorithm with survey data from December 2011 to August 2016 as a training dataset and from Sept 2016 to October 2018 as a testing dataset. The data comprised the locations and times of collection for presence and absence of HLB assessed by qPCR of leaf samples in commercial and residential locations (Fig 1B).

**Fig 4:**
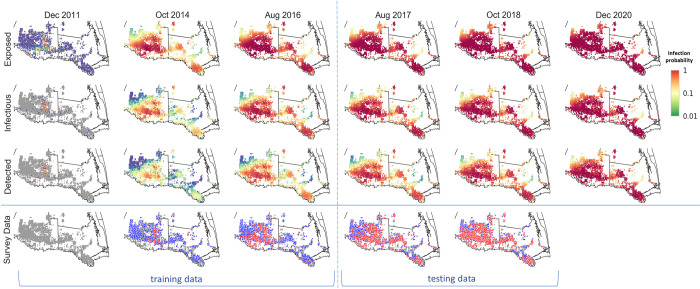
Spatiotemporal retrospective and prospective prediction of HLB spread in the Lower Rio Grande Valley, Texas. Spatiotemporal retrospective analysis of the historical spread of training period (Dec 2011 –Aug 2016) and prospective prediction of testing period (Sep 2016 –Oct 2018) and the future where no data were available (Nov 2018 –Dec 2020). We calculated infection risk by averaging over 1000 simulation realizations of the model fitted to the training data. Basemap shapefile for cartographic boundaries reproduced from U.S. Census Bureau under open data use.

The posterior estimates for the transmission rates (*ϵ*, *ε*_*B*_, *ε*_*W*_, *β*_*P*_, *β*_*V*_) and the dispersal scale (*α*) are summarised in Table 1, together with the average efficiency of the coordinated spraying program (*η*) adopted by commercial growers and the waiting period (*σ*^−1^) from being infectious to detected for a citrus grid cell. The corresponding marginal posterior distributions are summarised in S3 Fig. We observed good agreement between the model simulations and the training and testing data for both temporal and spatial validation metrics (Figs 3C, 3D and 4) with additional support from ROC curves for the model performance as a binary classifier that separated positive from negative cells (Fig 3B).

Inspection of the temporal curves for epidemic progress (Fig 3C) and retrospective mapping of the training data indicate an initial lag from the time that a cell becomes detectable according to the model and is recorded as detected by ground survey (Fig 4). Model simulations indicate that by October 2014, HLB had infected almost all of Hidalgo County (TX) whereas from the survey data it appeared as if the epidemic was just beginning to spread. The difference underlines the importance of cryptic (asymptomatic) infection in generating epidemic spread ahead of ground surveys [1,2,9]. Ground surveys, in turn, are frequently under-resourced during the early stages of epidemics, followed by an intensification in surveillance in response to increased awareness of the epidemic. Slow initial surveillance followed by acceleration in surveillance is consistent with the results in Figs 3 and 4).

**Fig 5:**
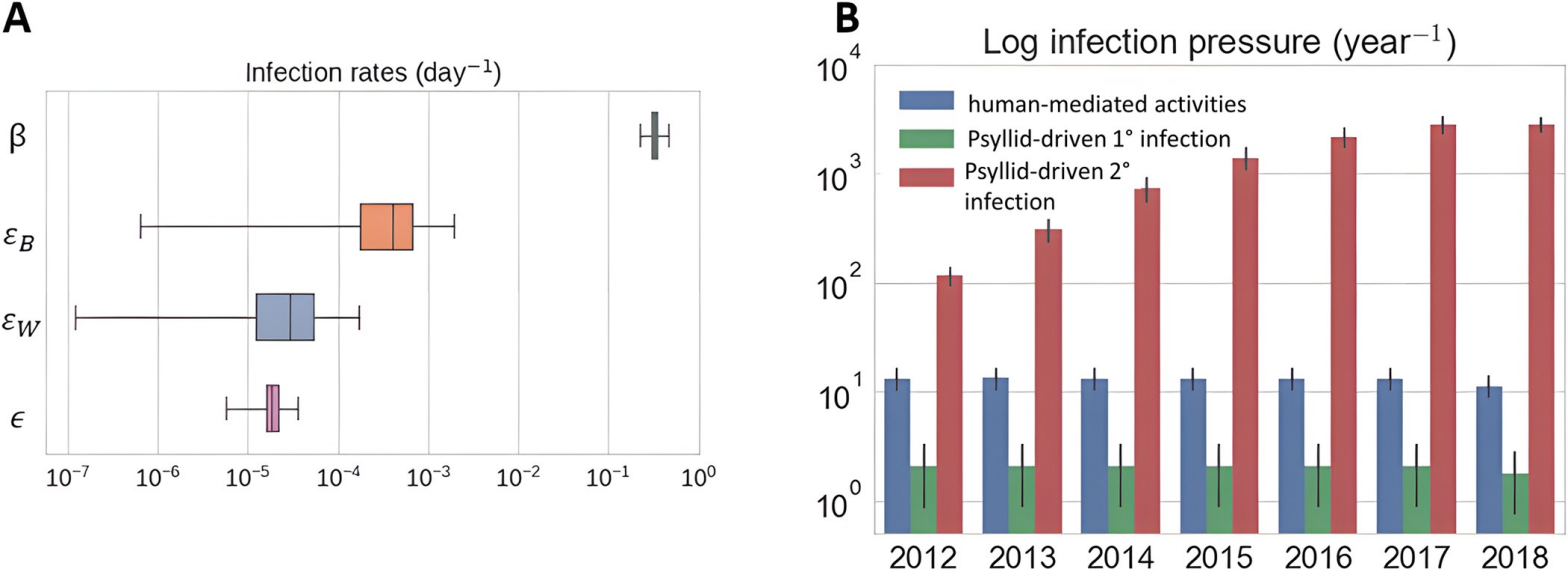
The role of primary and secondary transmission rates and forces of infection on HLB Spread in the Lower Rio Grande Valley, Texas HLB spread. (A) Posterior distribution of transmission rates via four sources: movement of local vectors from infectious trees in the landscape (β), the arrival of infected vectors over the international border (ε_B_) and from further away (ε_w_)and introduction of infected trees by other human-mediated movement (ε). (B) Contribution of the human-mediated, psyllid-driven primary infection and psyllid-driven secondary transmission sources to the realised infection pressure overall on susceptible trees in the survey period 2012–2018.

Local spread of HLB was dominated by secondary transmission involving ACP vectors (Fig 5A). The rate of secondary transmission was three orders of magnitude greater than the rates of primary infection via cross-border infected vectors and four orders larger than human-mediated movement of infected material (Fig 5A). We used an exponential prior for the cross-border infection rate (cf S4 Fig) to reflect the default belief that the rate is close to zero but we observed a clear departure from zero for the posterior distribution. We also found that the posterior estimate of longer distance (*ε*_*W*_) is much lower than immediate cross-border (*ε*_*B*_) primary transmission (Table 1). These results suggest that there are sources of infectious vectors close to the Mexico border. Fig 5B presents the annual infection pressure (calculated as the relevant components of *ϕ*_*i*_ (Eq 1 in Methods section) and summed over the whole landscape and each year) caused by the four infection sources. We again observe the significant role of local vector movement in driving the epidemic, resulting in infection pressures an order of magnitude larger than primary forces in early years, rising to two orders in the later years.

### Retrospective analysis of the effectiveness of the ACP control strategy in the Lower Rio Grande Valley in Texas

**Fig 6:**
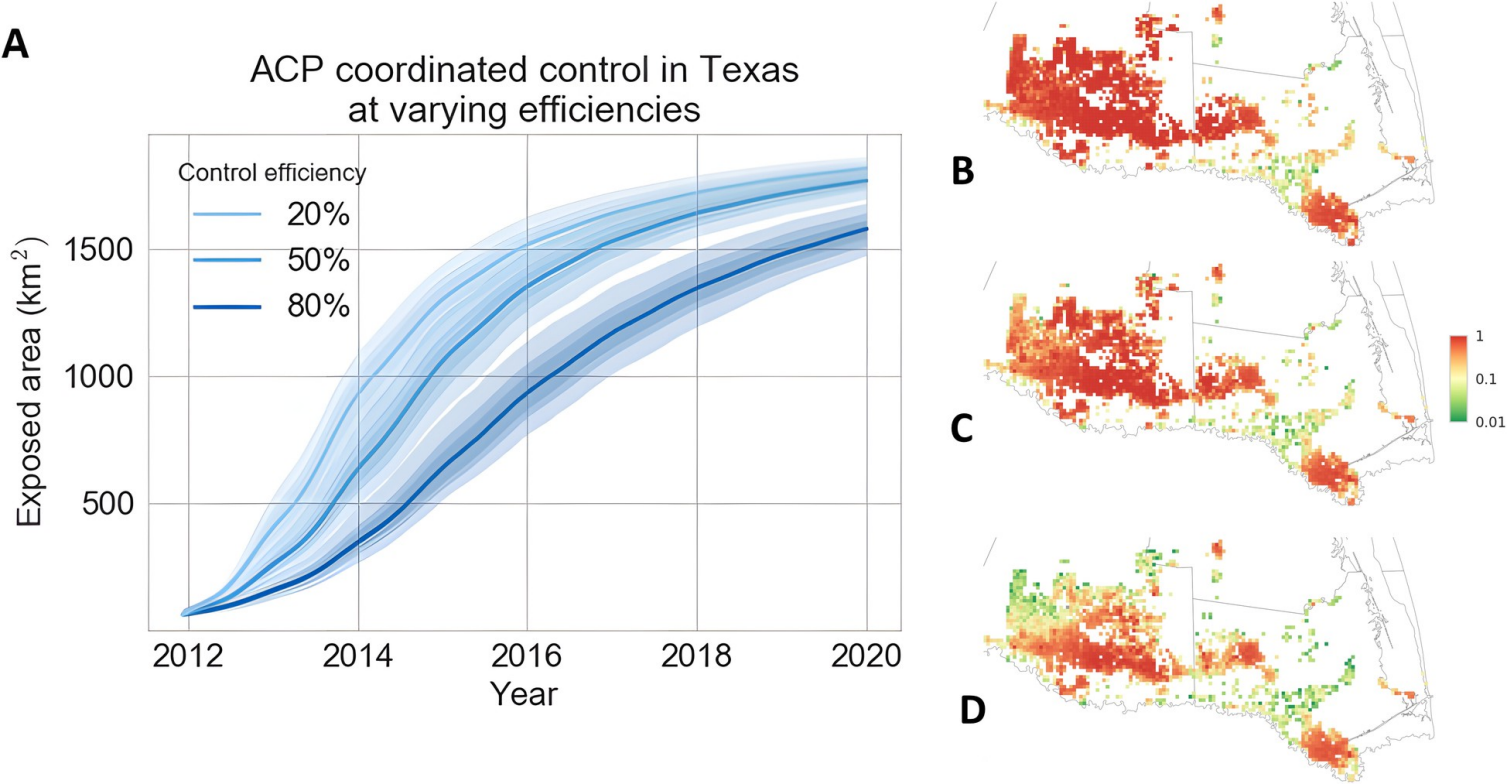
Retrospective analysis of the effectiveness of vector control scenarios in slowing down HLB spread. The effect of the efficiency of the annual coordinated spraying program on (A) the temporal progression and (B-D) spatial snapshots in October 2014 of the cumulative Exposed area. We considered three different hypothetical area-wide coordinated control scenarios at efficiencies of (B) 20%, (C) 50% and (D) 80%. Basemap shapefile for cartographic boundaries reproduced from U.S. Census Bureau under open data use.

The parameterised model enabled retrospective analysis of the area-wide coordinated vector spraying program in Texas. Beginning in 2011, growers joined the program by applying pesticide sprays within a short, designated period to target the overwintering vector populations. Using historic HLB survey data, we estimated that the program helped to reduce approximately 80% of the vector population in commercial orchards, designated as the control efficiency (Fig 6A). To understand the importance of having an area-wide collaborative effort amongst growers in place, we ran retrospective simulations for hypothetical scenarios in which less intensive ACP control had been carried out (Fig 6B and 6C). We observed a nonlinear relationship between the control efficiency and HLB exposed area. While increasing control efficiency from 20% to 50% did not guarantee the reduction of epidemic size, bringing control efficiency up to 80% was effective (we observed both a significant reduction of the infected area in 2020 and a clear separation of the 95% credible intervals associated with 20% and 80% control efficiency) (Fig 5A).

### Transfer of the model and prediction of HLB spread in southern California

The HLB epidemic in southern California was at an earlier stage compared with the outbreak in Texas, with clusters of infected trees found in the Orange and Riverside Counties (Fig 1E). Recorded HLB detections were limited to two clusters with intensive tree removal upon detection. Such intervention and the limited spatial representativeness of the data made it impossible to infer key parameters from the data. The data were sufficient, however, to test the credibility of transferring Texas parameters to this new region. We accounted for the difference in weather conditions that affect ACP distributions in California and Texas by incorporating a weather, suitability score for ACP growth, particularly for temperature [30] to the model. We also introduced HLB quarantines and the removal of HLB-confirmed trees as discrete stochastic events into the spread model.

**Fig 7:**
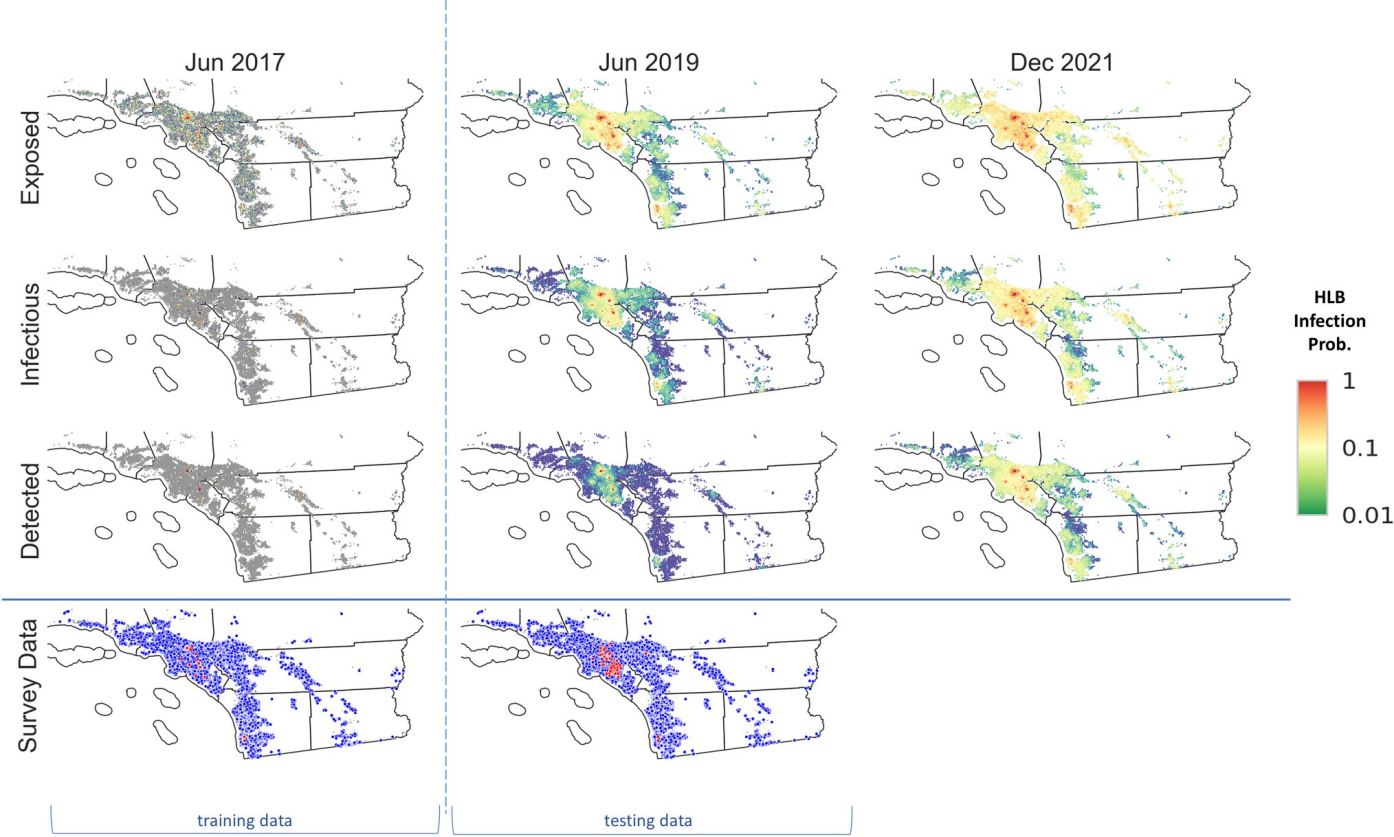
Spatiotemporal prediction of further HLB spread in southern California. We used California HLB survey data up to June 2017 to infer the locations of the hidden infectious cells and used the estimations to seed forward simulations. We calculated infection probabilities by averaging outputs from 1000 simulation runs for the prospective spread between June 2017 and December 2021. Basemap shapefile for cartographic boundaries reproduced from U.S. Census Bureau under open data use.

**Fig 8:**
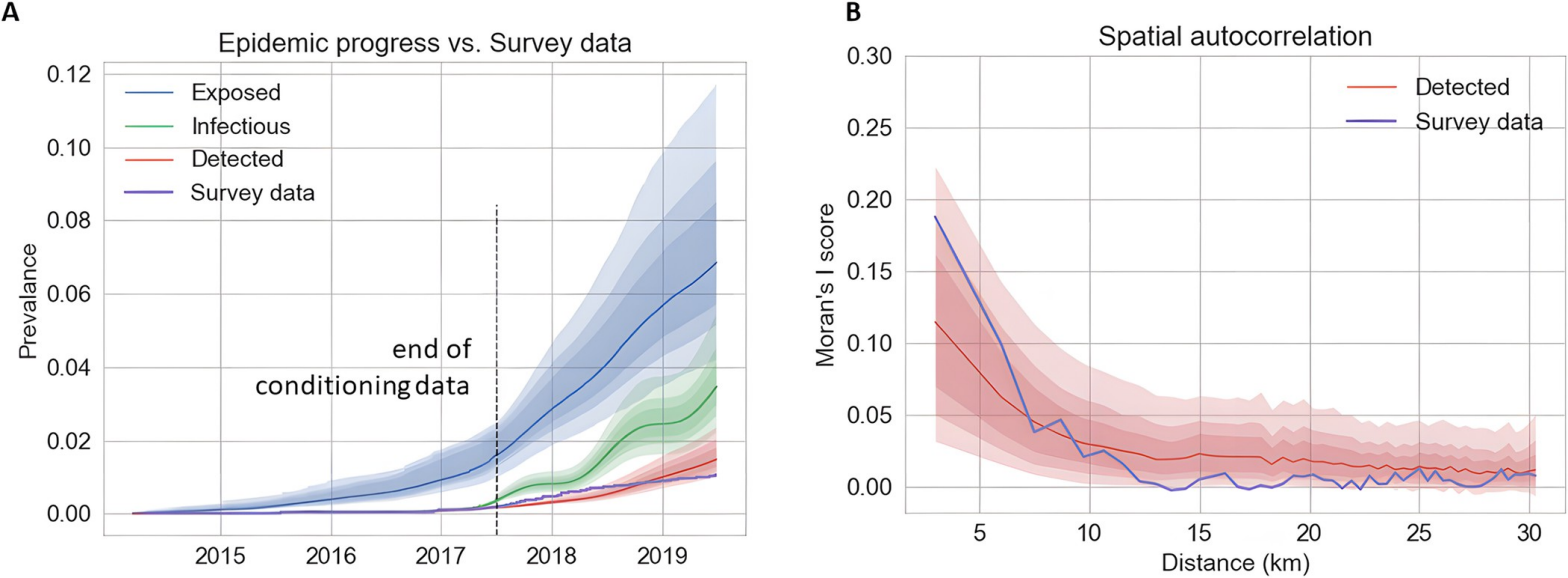
Evaluation of HLB epidemic progress in southern California vs survey data and the impact of quarantine radius to epidemic outcome. (A) Temporal progression of the prevalence of three infection categories (Exposed, Infectious, and Detected) in comparison with the training and testing data. We show means of 1000 simulation realizations as solid lines, and 50%, 75%, 95% credible intervals as shades of decreasing intensities. (B) Comparison of the spatial autocorrelation scores of the HLB Detected categories (red line and shades) in southern California with that of the HLB survey data (purple line) at the end of the testing data (June 2019).

We inferred the locations of unobserved infected cells up to 30^th^ June 2017 in southern California using the survey data collected before that date and thereafter simulated the epidemic forward. For the two years in the testing data (June 2017—June 2019), the predicted detections successfully reproduced the spatiotemporal patterns observed in the survey data (Fig 7). The predictions also showed good quantitative agreement with the training data and initially for the testing data for the temporal progression (Fig 8A) but while the model predicted a continued upward trend in the amount of detected grid cells, the surveillance data suggest a slowing towards a linear rate of increase. The observed measure of the spatial autocorrelation metric (Moran’s I) for the surveillance data lay within the credible intervals predicted by the model albeit with some subjective evidence of underestimation at short and overestimation at longer distances (Fig 8B). Inspection of the model predictions for unobservable infectious categories (exposed and infectious cells) indicated that the extent of HLB spread is likely to be far greater than was detected by survey data. Huanglongbing is likely to be present in most counties in southern California and even with the imposition of quarantines and tree removals upon detection, the disease was increasing in severity. Our results indicate that surveys by visual inspection are likely to reveal more HLB positive samples from all over Los Angeles and Orange Counties, and infected trees in San Bernardino, Riverside and San Diego Counties would become detectable.

### Effect of changing quarantine radius on disease management in southern Californian

To assess the effect of increasing the radius of quarantine circles to decrease the HLB infectious area in southern California, we used the counts of 1km^2^ cells that had been HLB infectious up to December 2021 as the evaluation metric. We started simulations in June 2017 using the inferred infected locations from data up to that point. We did not make use of either survey data or the quarantine boundaries data available after this time. In the absence of the availability of detailed information from the ground regarding the timings of removal and quarantine events after detection, we sample the lagging time as stochastic events. However, control events are systematic and deterministic in terms of location i.e. cells are marked for removal and quarantine if they or a nearby cell becomes detected with HLB. We evolved the boundaries of the quarantine area as the model predicted new detections. The model used the detection rate estimated from Texas. Besides the implementation of HLB quarantines, we also incorporated the effect of the annual coordinated spraying and the removal of HLB infected trees upon positive diagnostic confirmations.

**Fig 9:**
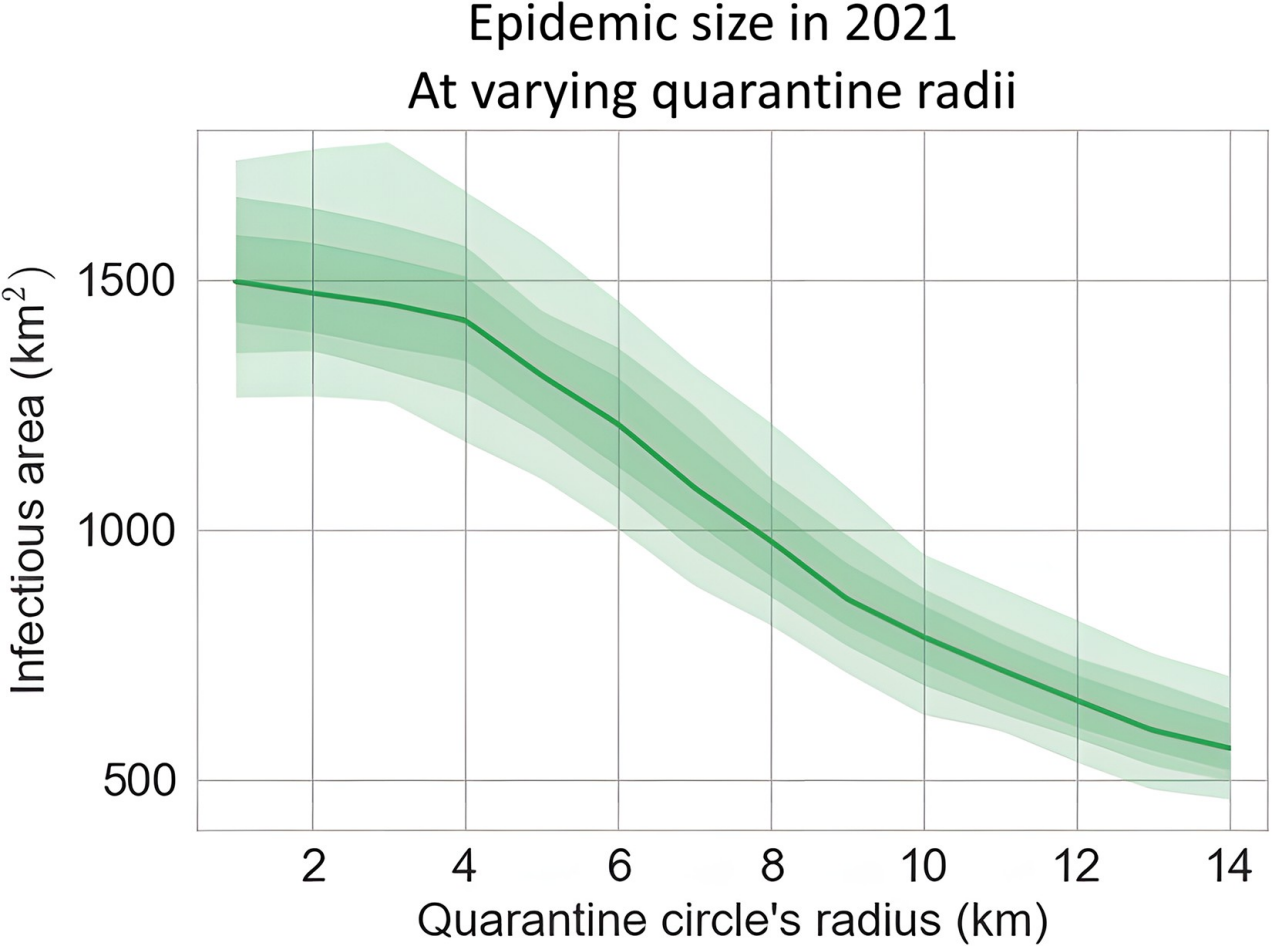
The effect of the radius of the quarantine area centring around HLB newly detected sites on the total Infectious area for southern California. We started simulations from June 2017 and assessed the total infectious area for December 2021.

We simulated 1,000 epidemic trajectories for each quarantine radius of 1, 2, 3, …, 14 km and derived the median and credible intervals from the generated samples. Simulation results demonstrated consistent reduction of the infectious regions as the quarantine radius is increased (Fig 9), with the then currently prescribed 8 km radius helping to reduce a third of the epidemic size by the end of 2021.

### Estimation of ACP invasion rate in the Central Valley using Texas ACP survey data

**Fig 10:**
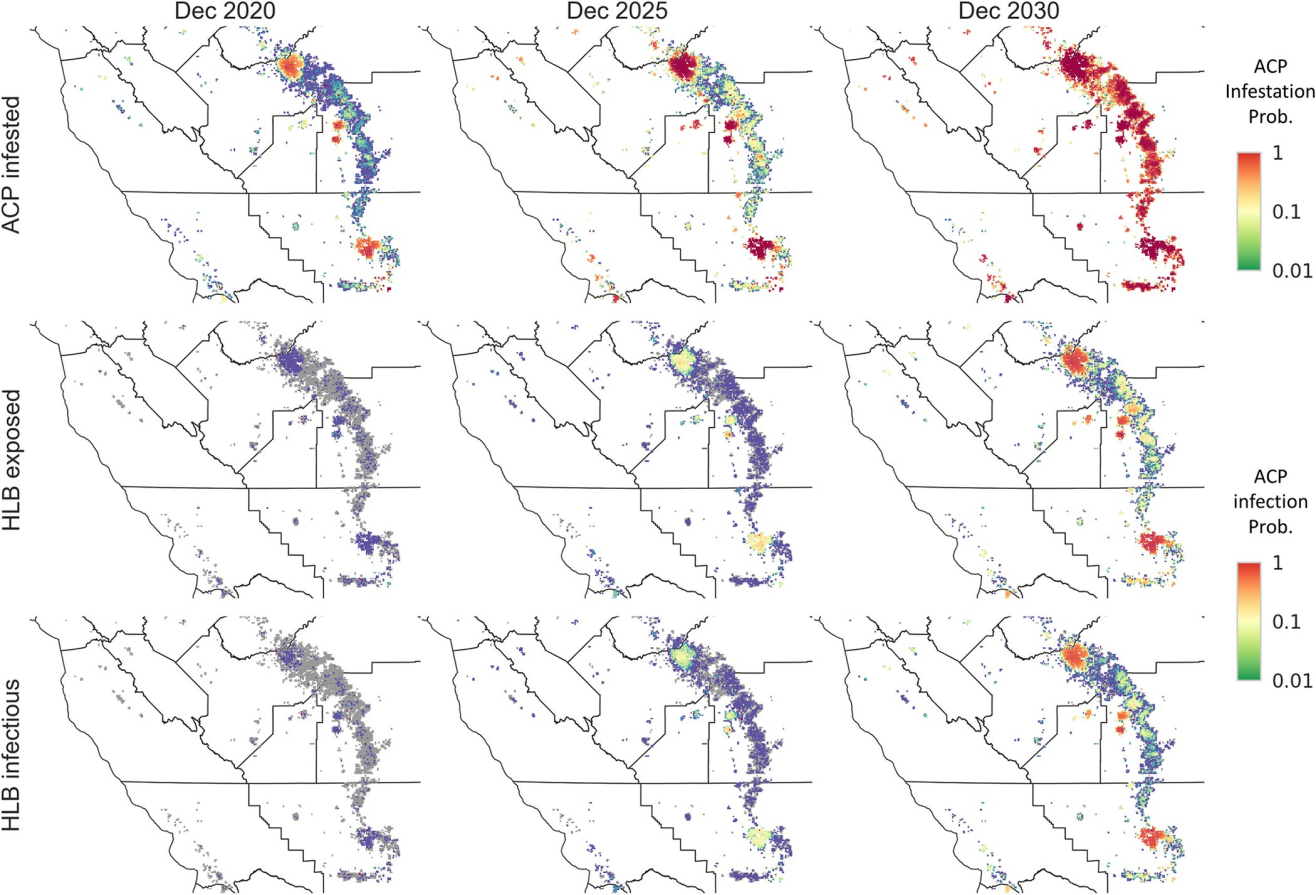
Spatiotemporal prediction of the potential ACP and HLB spread in the Central Valley. We used Central Valley ACP trapping data to seed the simulations for ACP spread and locations of inconclusive HLB samples (Ct value less than 38 in qPCR diagnostic test) as the initial infected HLB sites. HLB spread can only happen between ACP infested sites. We calculated the ACP infestation and HLB infection probabilities by averaging over 1000 simulation runs for the prospective epidemics from January 2020 to December 2030. Basemap shapefile for cartographic boundaries reproduced from U.S. Census Bureau under open data use.

The potential epidemic of HLB in the Central Valley, the major citrus growing area in California, was at a much earlier stage than for southern California, with the vector, ACP, invading rather than being established (Fig 1C and 1F). Modelling the spread for ACP at the landscape scale requires an estimate of the vector invasion rate. Exploratory analyses showed this was not possible using the small amount of ACP trapping data for the Central Valley: instead, we calculated the ACP invasion rate using the more extensive ACP survey dataset for the Lower Rio Grande Valley in Texas. Although ACP had fully invaded Texas by the start of data collection in 2011, the region was not fully infested with HLB-infected ACP. By introducing a new epidemic category ‘ACP + HLB infected’ (Fig 2B) that connected the dynamics of ACP infestation to HLB infection in a grid cell (S1A Fig), it was possible to estimate an invasion rate parameter for ACP from the ACP diagnostic data collected as part of the Texas HLB survey (S1B Fig). In particular, the ‘ACP + HLB infected’ category marked cells containing HLB-infected ACPs. By modelling the transition of cells from ‘ACP infested’ to ‘ACP + HLB infected’, we estimated the rate at which vectors move from one cell to another. Spatiotemporal maps for the potential spread of the ACP vector and HLB infection up to 2030 are shown in Fig 10 on the assumption of transferable parameters from Texas with suitable adaptation for weather conditions in the Central Valley.

**Fig 11:**
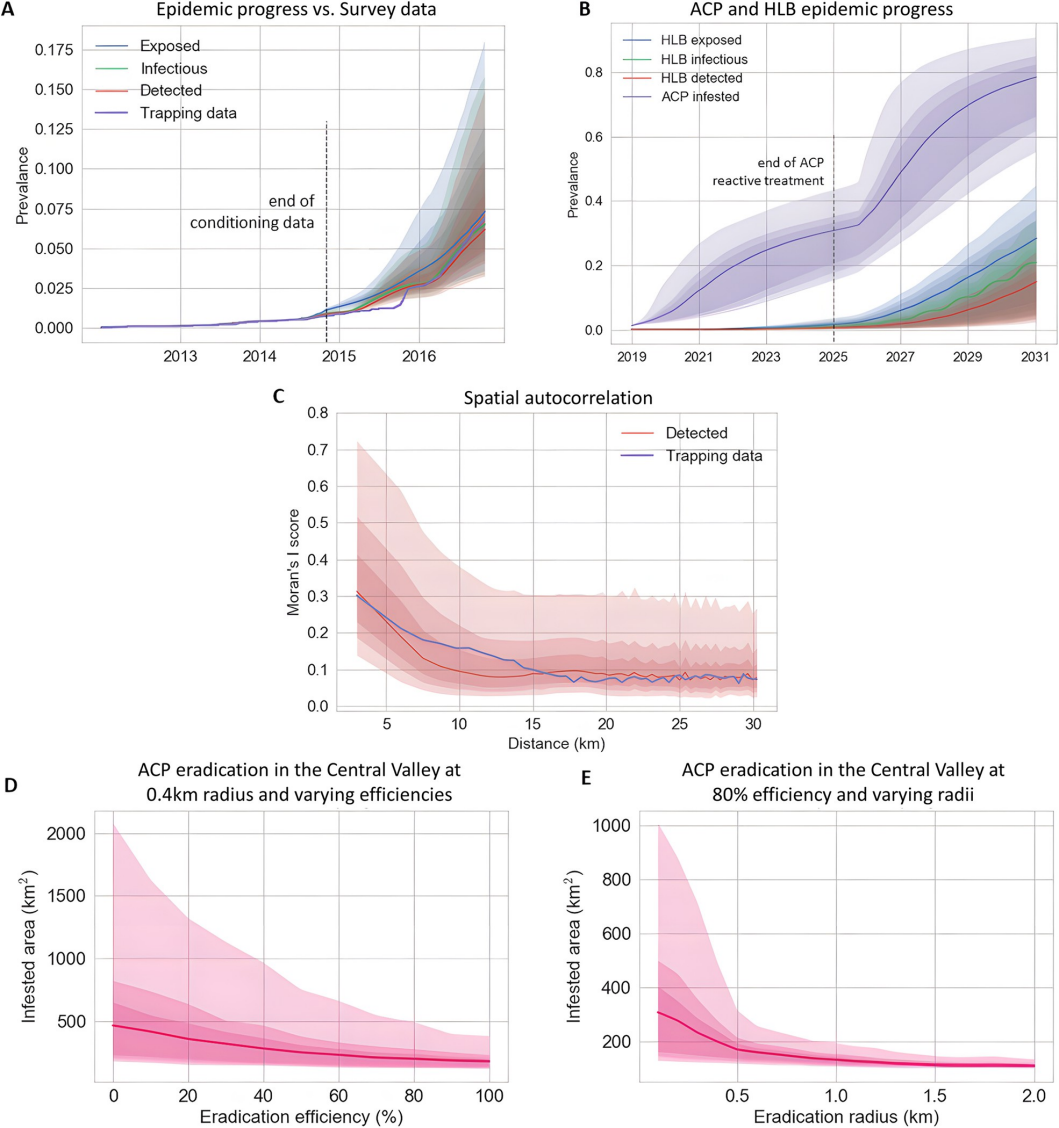
Potential ACP and HLB epidemic progress in the Central Valley and the likely effectiveness of control. (A) Temporal progression of the predicted infestation prevalence in comparison with the trapping data for the data availability period (up to October 2017). (B) Temporal progression of the predicted ACP and HLB epidemics from January 2019 to December 2030. We assumed that as ACP prevalence passes 0.3, ACP the reactive treatment program would have been dropped due to the high cost of maintaining the program and the reduced effectiveness as ACP becomes widespread. (C) Comparison of the spatial autocorrelation scores of the predicted ACP infestation prevalence (red line and shading) in the Central Valley with that of the ACP trapping data (purple line) at the end of the data availability period. (D,E) The effect of varying the efficiency and radius of pesticide treatment upon detection of the vector ACP on the total Infested area in the Central Valley. We started simulations from January 2020 and calculated the total Infested area for December 2021 for (D) control efficiency from 0% to 100% for treated circles of radius 0.4 km and (E) treatment radius from 0.1 km to 2 km assuming spraying efficiency of 80%.

We validated the ACP spread model by running simulations to reproduce the historic spread in the Central Valley in 2015 and 2016 and compared model predictions with the ACP trapping data, which were collected independently from the HLB survey and not used for parameter estimation. We incorporated the dynamics of the reactive ACP treatment program by the California Department of Food and Agriculture (CDFA) into the model and observed good agreement in both temporal progression (Fig 11A and 11B) and spatial autocorrelation metrics (Fig 11C), indicating that the ACP spread model successfully captures the ACP invasion dynamics.

### Potential efficiency of vector control in the Central Valley

As the focus for the Central Valley was on ACP invasion, we considered two parameters that drive a reactive ACP eradication program: the eradication efficiency (Fig 11D), and the radius of the treated circle around an ACP positive site (Fig 11E). Our simulations allowed for pesticide treatment, applied by CDFA, on all citrus trees within a 400 m radius of an ACP positive site. Where treatment circles overlap a commercial grove, the whole grove is treated. Reactive treatment occurs in addition to a coordinated spray implemented annually by commercial growers. Simulation results show that having both high efficiency and sufficient radius are essential to slow down the spread of ACP in the Central Valley (Fig 11D and 11E). There is a consistent reduction in the median infested area and also the 95% credible interval as the eradication efficiency increases. An eradication efficiency of 80% reduces the median infested area by half. It also reduces the upper boundary of the credible interval by 75% compared with no treatment. Increasing the eradication radius from 100 m to 500 m decrease the expected infested area by 50% and the credible interval by 75%. The results suggest that although 80% eradication efficiency is reasonable, it might be worthwhile to increase the treatment radius to 500 m.

## Discussion

Our primary aim in this paper was to develop and test an epidemiological modelling framework to predict the spread and control of Huanglongbing disease on citrus at extensive landscape scales (cf Fig 1). Our stochastic framework takes account of the intrinsic uncertainty of disease spread through heterogeneous citrus populations that encompass backyard trees and commercial citrus plantations. The framework was designed to be flexible. It allows for cryptic infection from sites that are infected but not yet symptomatic or detectable [1]. The model framework can be adapted to account for multiple sources of infection, and it has tuneable parameters that are adjustable to simulate a range of control scenarios. The models can also be adapted to allow for cases where the vector is endemic (Fig 2A) as in Texas and southern California or when the vector is also invading (Fig 2B) as in the Central Valley in California.

Having compared several model variants, the selected SEI model with an additional detected class (Fig 2A), an exponential dispersal kernel, normalised vector flux and allowance for vector control in commercial plantations successfully fitted the data for the citrus growing region in the Rio Grande Valley in Texas (Figs 3 and 4). Although sparse relative to the size of the host population, the surveillance data for Texas had the advantage over other datasets, such as for Florida, in that systematic surveys for both ACP and for Las had been conducted across a wide area before the first HLB positive trees were found. This allowed for direct observation of the spread of the disease from known points of introduction, which aided parameter estimation.

### Insights from model fitting on dynamics of HLB spread in Texas

Our results distinguish four potential transmission rates in the Rio Grande Valley (Fig 5). Secondary transmission by local movement of infected vectors is the dominant force of infection in epidemic spread following introduction (Fig 5B). We show, however, that longer-distance vector spread, local cross-border border and human-mediated movements all contribute to the initiation of new infected sites. Secondary transmission is remarkably effective in spreading the pathogen thereafter (Fig 5B). Moreover, retrospective inference of HLB infection times for Texas showed that the epidemic progressed at a much faster pace than had been captured by the survey data. There is a marked difference between the extent of spread of the detected class, which is comparable with the surveillance data, and the exposed and infectious classes (Fig 4). Knowledge of the locations of cryptically infected sites gives government and industry decision-makers a two-year advantage in knowledge of the extent of the epidemic when compared with survey data alone. Failure on the part of regulators and other stakeholders to allow for the enhanced spread of the pathogen beyond what is immediately detectable can lead to serious underestimation of the severity and impact of emerging epidemics [11].

We were able to infer the average efficiency of the annual coordinated spraying programme from the Texas survey data and to run retrospective analyses to compare with less effective uptake (Fig 6). Our results showed the benefit of having a high efficiency equivalent to a high level of participation from commercial growers in slowing epidemic progression, especially during the first few years after the invasion (Fig 6A). Thereafter, however, the benefit is insufficient to prevent the ultimate spread of the epidemic through the target region (Fig 6). Allowance for the localised disruption and interference of control on the intrinsic rates of epidemic spread is an important and often overlooked challenge for parameter estimation of emerging epidemics. Parry et al. [8] examined the impact of control on transmission and dispersal parameters for HLB at the plantation scale. Here we have extended the results from Parry et al. [8] using a similar SEI model and MCMC estimation to the landscape scale.

### Efficiency of MCMC estimation

Surveillance data that comprise incomplete snapshots of disease at successive times also pose a significant challenge for parameter estimation [7,8]. We used a Markov chain Monte Carlo method with data augmentation (DA-MCMC) to infer chains of infection. The DA-MCMC method is considered a robust approach for inferring parameters for stochastic, individual-based epidemiological models [8,32,38,39]. The method has been used to estimate epidemiological parameters for heterogeneous plantation-scale epidemic systems with cryptic infections [8]. Related applications of the methods beyond plant disease include foot-and-mouth outbreaks in cattle [38], avian influenza epidemics in poultry [40] and MRSA outbreaks in hospital wards [41].

Convergence of the DA-MCMC method, however, is known to be difficult when applied to domains (landscapes) with heterogeneously distributed target populations. Accordingly, we improved the mixing and convergence of MCMC samplers for unobserved epidemic transitions by utilising the randomised construction of Markov trajectories [34] and exact inference algorithms for hidden Markov models [35]. Using the improved MCMC samplers, it was possible to infer locations that were cryptically (i.e., asymptomatically) infected from the survey data available at the time of prediction. Initialising spatio-temporal epidemic models with asymptomatic as well as symptomatic infected sites was essential to capture the current extent and the future potential for disease spread.

### Transfer of the models to California

Transfer of the models to southern California gave encouraging results albeit in general patterns of epidemic spread, albeit with a caveat that some dynamics are not yet accounted. Allowing for cryptically infected sites (estimated from the training data) as well as survey reports of symptomatic sites when initialising the HLB spread model in southern California gave very good agreement in predicting the spread patterns observed in the two years of test data (Fig 7). There was also good agreement between model predictions and data for the spatial autocorrelation metric (Fig 8B). Dynamic risk maps for HLB in southern California from the epidemiological model (Fig 7) were markedly consistent with the risk maps derived by McRoberts et al. [24] from a statistical model. Building on earlier work to analyse risk in Florida [42] the statistical model used a mixture of social, biophysical and environmental variables to quantify risks across a rasterised landscape (1.6 km^2^) analogous to the 1km^2^ grid cells in the epidemiological model. Narouei-Khandan et al. [43] also used regression methods to relate global occurrence of HLB with historic climate data. The models also predicted that coastal areas in California were climatologically favorable for ACP and Las, but less so than in Florida. When current USA presence data were included in the models, the suitable areas for ACP establishment expanded to the Central Valley, CA, while this area remained less conducive for Las.

Careful inspection of the future predictions of the temporal data for southern California (Fig 8A) indicated evidence of deviation between the survey data and the detected class during the test period. The model results predicted continued acceleration in the rate of disease spread compared with a near-linear rate of increase in the surveillance data up to June 2019 (Fig 8A). Expert opinion based upon ongoing sampling of the vector indicates a striking difference between Texas and California in the percentage of ACP samples testing positive for Las. Whereas >50% of psyllid samples tested positive for Las in Texas by 2018, only 0.25% of psyllid samples have tested positive for Las in California since 2017 (Bartels, pers comm). The highest level (0.6%) in southern California was reached in the 2^nd^ quarter of 2022. Even when data from the core areas of HLB positive trees are separated out, the percent ACP samples positive for Las is ~2% of the total. The proportion of infested psyllids in Texas is comparable with reports from other states and countries, albeit with sometimes considerable variation. Wulff et al. [44] found the incidence of ACP with Las ranged from 33 to 74.6% in Brazil’s citrus belt, while Hall [45] reports a mean of 17.5% of ACP with Las in Florida. Hu et al. [46] observed found the incidence of ACP with Las in China ranged from 3 to 78% and was correlated with HLB incidence in citrus trees.

The reason for the difference in the uptake of Las by ACP in California compared with other areas is unknown and requires further study to determine how the strains of Las in California interact with the ACP vector. Four different strains of Las in California, based on prophage typing groups, have been detected with sequence data indicating that the California strains were not introduced from Florida but are likely to have come from Asia [47]. Our model was adjusted to account for differences in temperature patterns between southern California and Texas, with essentially no vector dynamics outside 10° - 33°C [30] in California. This might underestimate survival in certain high temperature inland regions. Antolínez et al. [48] recently analysed and discussed heat and dry stress on ACP, noting that survivorship at high temperatures is likely to depend upon exposure time. Antolínez et al. [48] suggest this may account for low ACP numbers during very hot summer months in inland areas of southern California compared with relatively higher ACP numbers reported for non-desert areas close to the coast.

Heat and dry stress are not sufficient to account for low uptake of Las by ACP and subsequent effects on transmission of Las. Our model parameters for the rates of acquisition of Las by ACP (ξ), and of primary (ε_V_) and secondary infestation (β_V_) can be altered to accommodate changes in uptake, with β_P_ adjusted for transmission rates. This, however, requires further data and analysis.

### Provisional control scenarios in California

The models were used to investigate the impact of different control strategies on epidemic outcomes for HLB in California under an assumption of the ability of the vector to acquire and transmit infection. While necessarily speculative, the analyses indicate the likely intrinsic sensitivity to adjusting the intensity of quarantine. Increasing the radius of the quarantine area to prevent movement of citrus products around newly detected HLB sites has the potential to reduce the total Infectious area for southern California (Fig 9). Within the Central Valley, our results indicate that changing the radius and the efficiency for the reactive ACP treatment programme could each reduce the infested area (Fig 11A and 11B) if a significant epidemic were to occur. There was also a marked reduction in the uncertainty of the outcomes of the programmes with enhanced control effort (Fig 11A and 11B).

### Predictive models for disease outbreaks

Predictive models are an important tool in the management of infectious disease outbreaks. Here we have used compartmental epidemiological models coupled with dispersal kernels to analyse and the predict the spread of HLB. Epidemiological models of this type have the advantage that both the state variables (e.g. susceptible, exposed, infectious sites) and the parameters (e.g. transmission rates, time to detection, dispersal kernel) have intrinsic biological meanings [1].The models can also be readily adapted to incorporate mechanisms for control, as here, for quarantine and vector control. Parry et al. [8], Chiyaka et al. [49], Neri et al. [7] used compartmental frameworks to model the dynamics of citrus disease at scales ranging from individual plants [49] through plantations [8] to local landscapes [7]. Parry et al. [8] and Neri et al. [7] also addressed fundamental issues in the use and analysis of Bayesian methods to estimate parameters from surveillance data for emerging epidemics of HLB and citrus canker. Haynes et al. [50] used a related approach of agent-based modelling to analyse ACP and HLB spread on a lattice of nine plantations. Our work, in this paper, has extended the scale to analyse and predict the spread and control of the pathogen and the vector at large heterogeneous landscape scales that extend across multiple counties. We also address the transferability and modulation of parameter estimates derived from one region to other regions. Compartmental epidemiological models, analogous to those introduced here, have also been widely used to respond to outbreaks and to formulate current and future policies for livestock and human diseases. Examples range from early work on foot-and-mouth disease [26,51], severe acute respiratory syndrome (SARS) [52] and recent intensive work on SARS-CoV-2 (e.g. [53–55]). Parameter estimation for emerging epidemics of human and livestock populations frequently benefits from enhanced reporting and more extensive mapping of outbreaks than is usual for crop disease. Many of the challenges, however, in extracting signals from comparatively sparse data are common to all types of infectious disease epidemiology and deserve more study to assess how soon and for what types of data robust initial estimates of epidemiological parameters can be obtained for emerging epidemics. Statistical methods, based on regression and correlation, along with the increasing popularity of machine learning [56] offer alternative approaches to assessing disease risk. McRoberts et al. [24] estimated landscape-scale risk maps for HLB in California at an analogous spatial resolution to the one used in this paper while Alves et al. [57] used a hierarchical Bayesian modelling approach to link climatic variables with the regional prevalence of HLB (at a spatial resolution of 55 x 55 km) in Minas Gerais state in Brazil. Further work is needed to assess the relative merits and complementarities of different approaches to modelling risk and assessing options for control of emerging epidemics such as for HLB on citrus.

## Supporting information

S1 Text Technical Appendix. Contains additional details of data, epidemiological models and for methods parameter estimation and model prediction used in the analyses.
(DOCX)

S1 Fig The epidemiological model and Texas data used to estimate the ACP invasion rate. (**A**) The joint model of ACP and HLB spread after accounting for the fact that the vector had fully infested the citrus landscape before the modelling period. The ACP and HLB spread share the dispersal scale parameter and differ by the rates of invasion *β*_*V*_ and transmission *β*_*P*_ respectively. *τ*_*V*→*H*_ denotes the probability or efficacy of pathogen transmission to trees during the feeding of infected psyllids. The full model which allows for emerging ACP population is shown in Fig 2B (main text). (**B**) Geo-coded diagnostic samples of the vector ACP collected between December 2011 and October 2018 as part of the HLB state-wide survey in Texas. Samples with Ct value less than 38 were marked as positives. Diagnostic samples of citrus leaves collected as part of the same survey are mapped in Fig 1D (main text).
(TIF)

S2 Fig Evaluation of temporal progression for the predicted HLB detections in Texas against survey data for alternative models. We considered the temporal progression of the prevalence of three infection categories (Exposed, Infectious, and Detected) for the HLB epidemiological model and its four model variants. Each model variant removes one key component from the HLB epidemiological model used throughout the paper. We show means of 500 simulation realizations as solid lines, and 50%, 75%, 95% credible intervals as shades of decreasing intensities. (**A**) The full HLB epidemiological model as described in the Methods section. (**B**) No normalisation, in which the normalisation term for vector fluxes is assumed to be the same for all cells and absorbed into the secondary infection rate. (**C**) No control effect, in which we ignored the occurrence of the annual coordinated spraying program. (**D**) No border effect, in which we did not distinguish between sites near to and far from the Mexico border and used the same primary infection rate for infected vector from external environments. (**E**) power-law kernel, in which the exponential dispersal function is replaced with a power-law function.
(TIF)

S3 Fig Evaluation of spatial metrics for the predicted HLB detections in Texas against survey data for alternative models. We considered the spatial autocorrelation scores of the Detected categories (green line and shades) with respect to the survey data (purple line) at the end of August 2017 for the HLB epidemiological model and its four model variants. (**A**) The full HLB epidemiological model as described in the Methods section. (**B**) No normalisation, in which the normalisation term for vector fluxes is assumed to be the same for all cells and absorbed to the secondary infection rate. (**C**) No control effect, in which we ignored the occurrence of the annual coordinated spraying program. (**D**) No border effect, in which we did not distinguish between sites near to and far from the Mexico border and used the same primary infection rate for infected vector from external environments. (**E**) power-law kernel, in which the exponential dispersal function is replaced with a power-law function.
(TIF)

S4 Fig **Posterior distributions of key parameters for HLB epidemic model** in comparison with uninformative prior distributions (dotted black line). Legend numbers give the mean posterior value and the 95% credible interval for each parameter.
(TIF)

## References

[1] Gilligan CA. Sustainable agriculture and plant diseases: An epidemiological perspective. Phil Trans R Soc B. 2008;363: 741–759. doi: 10.1098/rstb.2007.2181 17827101 PMC2610107

[2] Gilligan CA, van den Bosch F. Epidemiological models for invasion and persistence of pathogens. Annu Rev Phytopathol. 2008;46: 385–418. doi: 10.1146/annurev.phyto.45.062806.094357 18680429

[3] Meentemeyer RK, Cunniffe NJ, Cook AR, Filipe JAN, Hunter RD, Rizzo DM, et al. Epidemiological modeling of invasion in heterogeneous landscapes: Spread of sudden oak death in California (1990–2030). Ecosphere. 2011;2: art17. doi: 10.1890/ES10-00192.1

[4] Wu JT, Leung K, Leung GM. Nowcasting and forecasting the potential domestic and international spread of the 2019-nCoV outbreak originating in Wuhan, China: a modelling study. The Lancet. 2020;395: 689–697. doi: 10.1016/S0140-6736(20)30260-9 32014114 PMC7159271

[5] Zhuang Z, Zhao S, Lin Q, Cao P, Lou Y, Yang L, et al. Preliminary estimates of the reproduction number of the coronavirus disease (COVID-19) outbreak in Republic of Korea and Italy by 5 March 2020. International Journal of Infectious Diseases. 2020;95: 308–310. doi: 10.1016/j.ijid.2020.04.044 32334115 PMC7194543

[6] Adrakey HK, Streftaris G, Cunniffe NJ, Gottwald TR, Gilligan CA, Gibson GJ. Evidence-based controls for epidemics using spatio-temporal stochastic models in a Bayesian framework. J R Soc Interface. 2017;14: 20170386. doi: 10.1098/rsif.2017.0386 29187634 PMC5721149

[7] Neri FM, Cook AR, Gibson GJ, Gottwald TR, Gilligan CA. Bayesian analysis for inference of an emerging epidemic: citrus canker in urban landscapes. PLoS Comput Biol. 2014;10: e1003587. doi: 10.1371/journal.pcbi.1003587 24762851 PMC3998883

[8] Parry M, Gibson GJ, Parnell S, Gottwald TR, Irey MS, Gast TC, et al. Bayesian inference for an emerging arboreal epidemic in the presence of control. Proc Natl Acad Sci USA. 2014;111: 6258–6262. doi: 10.1073/pnas.1310997111 24711393 PMC4035939

[9] Cunniffe NJ, Stutt ROJH, DeSimone RE, Gottwald TR, Gilligan CA. Optimising and communicating options for the control of invasive plant disease when there is epidemiological uncertainty. PLoS Comput Biol. 2015;11: e1004211. doi: 10.1371/journal.pcbi.1004211 25874622 PMC4395213

[10] Filipe JAN, Cobb RC, Meentemeyer RK, Lee CA, Valachovic YS, Cook AR, et al. Landscape epidemiology and control of pathogens with cryptic and long-distance dispersal: Sudden oak death in northern californian forests. Smith DL, editor. PLoS Comput Biol. 2012;8: e1002328. doi: 10.1371/journal.pcbi.1002328 22241973 PMC3252276

[11] Cunniffe NJ, Cobb RC, Meentemeyer RK, Rizzo DM, Gilligan CA. Modeling when, where, and how to manage a forest epidemic, motivated by sudden oak death in California. Proc Natl Acad Sci USA. 2016;113: 5640–5645. doi: 10.1073/pnas.1602153113 27140631 PMC4878485

[12] Taylor RA, Mordecai EA, Gilligan CA, Rohr JR, Johnson LR. Mathematical models are a powerful method to understand and control the spread of Huanglongbing. PeerJ. 2016;4: e2642. doi: 10.7717/peerj.2642 27833809 PMC5101597

[13] Kissler SM, Tedijanto C, Goldstein E, Grad YH, Lipsitch M. Projecting the transmission dynamics of SARS-CoV-2 through the postpandemic period. Science. 2020;368: 860–868. doi: 10.1126/science.abb5793 32291278 PMC7164482

[14] Gottwald T.R. Current epidemiological understanding of citrus huanglongbing. Annu Rev Phytopathol. 2010;48: 119–39. doi: 10.1146/annurev-phyto-073009-114418 20415578

[15] Parnell S, van den Bosch F, Gottwald T, Gilligan CA. Surveillance to inform control of emerging plant diseases: an epidemiological perspective. Annu Rev Phytopathol. 2017;55: 591–610. doi: 10.1146/annurev-phyto-080516-035334 28637378

[16] Sétamou M, Alabi OJ, Kunta M, Dale J, da Graça JV. Distribution of *Candidatus* Liberibacter asiaticus in citrus and the Asian citrus psyllid in Texas over a decade. Plant Disease. 2020;104: 1118–1126. doi: 10.1094/PDIS-08-19-1779-RE 32040392

[17] Grafton-Cardwell EE. Management of Asian citrus psyllid in California. In: Qureshi JA, Stansly PA, editors. Asian citrus psyllid: Biology, ecology and management of the Huanglongbing vector. CAB International; 2020. pp. 250–257.

[18] CDFA. Huanglongbing (HLB) regulatory and quarantine boundaries. California Department of Food and Agriculture; 2023. Available: https://www.cdfa.ca.gov/citrus/pests_diseases/hlb/regulation.html

[19] Warnert J. Asian citrus psyllid and huanglongbing disease threaten California citrus. Calif Agri. 2012;66: 127–130.

[20] Filho AB, Inoue-Nagata AK, Bassanezi RB, Belasque J, Amorim L, Macedo MA, et al. The importance of primary inoculum and area-wide disease management to crop health and food security. Food Sec. 2016;8: 221–238. doi: 10.1007/s12571-015-0544-8

[21] Cocuzza GEM, Alberto U, Hernández-Suárez E, Siverio F, Di Silvestro S, Tena A, et al. A review on *Trioza erytreae* (African citrus psyllid), now in mainland Europe, and its potential risk as vector of huanglongbing (HLB) in citrus. J Pest Sci. 2017;90: 1–17. doi: 10.1007/s10340-016-0804-1

[22] Bové JM. Huanglongbing: A destructive, newly-emerging, century-old disease of citrus. J Plant Pathol. 2006;88: 7–37.

[23] Ajene IJ, Khamis FM, van Asch B, Pietersen G, Seid N, Rwomushana I, et al. Distribution of *Candidatus* Liberibacter species in eastern Africa, and the first report of *Candidatus* Liberibacter asiaticus in Kenya. Sci Rep. 2020;10: 3919. doi: 10.1038/s41598-020-60712-0 32127552 PMC7054587

[24] McRoberts N, Figuera SG, Olkowski S, McGuire B, Luo W, Posny D, et al. Using models to provide rapid programme support for California’s efforts to suppress Huanglongbing disease of citrus. Phil Trans R Soc B. 2019;374: 20180281. doi: 10.1098/rstb.2018.0281 31104609 PMC6558569

[25] Parnell S, Gottwald TR, van den Bosch F, Gilligan CA. Optimal strategies for the eradication of asiatic citrus canker in heterogeneous host landscapes. Phytopathology. 2009;99: 1370–1376. doi: 10.1094/PHYTO-99-12-1370 19900003

[26] Keeling MJ, Woolhouse MEJ, Shaw DJ, Matthews L, Chase-Topping M, Haydon DT, et al. Dynamics of the 2001 UK foot and mouth epidemic: Stochastic dispersal in a heterogeneous landscape. Science. 2001;294: 813–817. doi: 10.1126/science.1065973 11679661

[27] Bayles BR, Thomas SM, Simmons GS, Grafton-Cardwell EE, Daugherty MP. Spatiotemporal dynamics of the Southern California Asian citrus psyllid (*Diaphorina citri*) invasion. Wang Z, editor. PLoS ONE. 2017;12: e0173226. doi: 10.1371/journal.pone.0173226 28278188 PMC5344380

[28] Gottwald T, Luo W, Posny D, Riley T, Louws F. A probabilistic census-travel model to predict introduction sites of exotic plant, animal and human pathogens. Phil Trans R Soc B. 2019;374: 20180260. doi: 10.1098/rstb.2018.0260 31104596 PMC6558561

[29] Li W, Li D, Twieg E, Hartung JS, Levy L. Optimized quantification of unculturable *Candidatus* Liberibacter spp. in host plants using real-time PCR. Plant Disease. 2008;92: 854–861. doi: 10.1094/PDIS-92-6-0854 30769724

[30] Liu YH, Tsai JH. Effects of temperature on biology and life table parameters of the Asian citrus psyllid, *Diaphorina citri* Kuwayama (Homoptera: Psyllidae). Ann Applied Biology. 2000;137: 201–206. doi: 10.1111/j.1744-7348.2000.tb00060.x

[31] O’Neill PD, Roberts GO. Bayesian inference for partially observed stochastic epidemics. Journal of the Royal Statistical Society: Series A (Statistics in Society). 1999;162: 121–129. doi: 10.1111/1467-985X.00125

[32] Gibson G, Renshaw E. Estimating parameters in stochastic compartmental models using Markov chain methods. Mathematical Medicine and Biology. 1998;15: 19–40. doi: 10.1093/imammb/15.1.19

[33] Chib S, Greenberg E. Understanding the Metropolis-Hastings algorithm. The American Statistician. 1995;49: 327–335. doi: 10.1080/00031305.1995.10476177

[34] Gross D, Miller DR. The randomization technique as a modeling tool and solution procedure for transient Markov processes. Operations Research. 1984;32: 343–361. doi: 10.1287/opre.32.2.343

[35] Rabiner L, Juang B. An introduction to hidden Markov models. IEEE ASSP Mag. 1986;3: 4–16. doi: 10.1109/MASSP.1986.1165342

[36] Moran PAP. Notes on continuous stochastic phenomena. Biometrika. 1950;37: 17–23. doi: 10.2307/2332142 15420245

[37] Upton GJG, Fingleton B. Spatial data analysis by example, volume 1: Point patterns and quantitative data. Chichester: John Wiley; 1985.

[38] Jewell CP, Keeling MJ, Roberts GO. Predicting undetected infections during the 2007 foot-and-mouth disease outbreak. J R Soc Interface. 2009;6: 1145–1151. doi: 10.1098/rsif.2008.0433 19091686 PMC2817150

[39] Keeling MJ, Hill EM, Gorsich EE, Penman B, Guyver-Fletcher G, Holmes A, et al. Predictions of COVID-19 dynamics in the UK: Short-term forecasting and analysis of potential exit strategies. Flegg JA, editor. PLoS Comput Biol. 2021;17: e1008619. doi: 10.1371/journal.pcbi.1008619 33481773 PMC7857604

[40] Jewell CP, Kypraios T, Christley RM, Roberts GO. A novel approach to real-time risk prediction for emerging infectious diseases: A case study in Avian Influenza H5N1. Preventive Veterinary Medicine. 2009;91: 19–28. doi: 10.1016/j.prevetmed.2009.05.019 19535161

[41] Kypraios T O’Neill PD, Huang SS, Rifas-Shiman SL, Cooper BS. Assessing the role of undetected colonization and isolation precautions in reducing methicillin-resistant *Staphylococcus aureus* transmission in intensive care units. BMC Infect Dis. 2010;10: 29. doi: 10.1186/1471-2334-10-29 20158891 PMC2829569

[42] Gottwald T.R., Luo W, McRoberts N. Risk-based residential HLB/ACP survey for California, Texas, and Arizona. In: Plant Management Network, American Phytopathological Society. [Internet]. 2013 [cited 24 Jan 2023]. Available: https://stream.cadmore.media/player/5c99e57c-c5fc-477c-8def-08d13f584984

[43] Narouei-Khandan HA, Halbert SE, Worner SP, van Bruggen AHC. Global climate suitability of citrus huanglongbing and its vector, the Asian citrus psyllid, using two correlative species distribution modeling approaches, with emphasis on the USA. Eur J Plant Pathol. 2016;144: 655–670. doi: 10.1007/s10658-015-0804-7

[44] Wulff NA, Daniel B, Sassi RS, Moreira AS, Bassanezi RB, Sala I, et al. Incidence of *Diaphorina citri* Carrying *Candidatus* Liberibacter asiaticus in Brazil’s citrus belt. Insects. 2020;11: 672. doi: 10.3390/insects11100672 33022967 PMC7650542

[45] Hall DG. Incidence of “*Candidatus* Liberibacter asiaticus” in a Florida population of Asian citrus psyllid. Journal of Applied Entomology. 2018;142: 97–103. doi: 10.1111/jen.12466

[46] Hu Y, Meng Y, Yao L, Wang E, Tang T, Wang Y, et al. Citrus Huanglongbing correlated with incidence of *Diaphorina citri* carrying *Candidatus* Liberibacter asiaticus and citrus phyllosphere microbiome. Frontiers in Plant Science. 2022;13. Available: https://www.frontiersin.org/articles/10.3389/fpls.2022.964193 36466264 10.3389/fpls.2022.964193 PMC9716883

[47] Dai Z, Wu F, Zheng Z, Yokomi R, Kumagai L, Cai W, et al. Prophage Diversity of ‘ *Candidatus* Liberibacter asiaticus’ Strains in California. Phytopathology. 2019;109: 551–559. doi: 10.1094/PHYTO-06-18-0185-R 30303769

[48] Antolínez CA, Olarte-Castillo XA, Martini X, Rivera MJ. Influence of daily temperature maximums on the development and short-distance movement of the Asian citrus psyllid. Journal of Thermal Biology. 2022;110: 103354. doi: 10.1016/j.jtherbio.2022.103354 36462881

[49] Chiyaka C, Singer BH, Halbert SE, Morris JG, van Bruggen AHC. Modeling huanglongbing transmission within a citrus tree. Proceedings of the National Academy of Sciences. 2012;109: 12213–12218. doi: 10.1073/pnas.1208326109 22783015 PMC3409777

[50] Haynes S, Singh A, Kaplan JD. An agent based model of ACP/HLB in California citrus—Preliminary results on the effects of insecticide and coordination on the spread of HLB. 2021; 4. Available: https://www.csus.edu/faculty/k/kaplanj/researchnotes/researchnote2021-2.pdf

[51] Ferguson NM, Donnelly CA, Anderson RM. The foot-and-mouth epidemic in Great Britain: Pattern of spread and impact of interventions. Science. 2001;292: 1155–1160. doi: 10.1126/science.1061020 11303090

[52] Riley S, Fraser C, Donnelly CA, Ghani AC, Abu-Raddad LJ, Hedley AJ, et al. Transmission dynamics of the etiological agent of SARS in Hong Kong: Impact of public health interventions. Science. 2003;300: 1961–1966. doi: 10.1126/science.1086478 12766206

[53] Kretzschmar ME, Ashby B, Fearon E, Overton CE, Panovska-Griffiths J, Pellis L, et al. Challenges for modelling interventions for future pandemics. Epidemics. 2022;38: 100546. doi: 10.1016/j.epidem.2022.100546 35183834 PMC8830929

[54] Marion G, Hadley L, Isham V, Mollison D, Panovska-Griffiths J, Pellis L, et al. Modelling: Understanding pandemics and how to control them. Epidemics. 2022;39: 100588. doi: 10.1016/j.epidem.2022.100588 35679714

[55] Stutt ROJH, Retkute R, Bradley M, Gilligan CA, Colvin J. A modelling framework to assess the likely effectiveness of facemasks in combination with ‘lock-down’ in managing the COVID-19 pandemic. Proc R Soc A. 2020;476: 20200376. doi: 10.1098/rspa.2020.0376 32821237 PMC7428039

[56] Parselia E, Kontoes C, Tsouni A, Hadjichristodoulou C, Kioutsioukis I, Magiorkinis G, et al. Satellite earth observation data in epidemiological modeling of malaria, dengue and West Nile virus: a scoping review. Remote Sensing. 2019;11: 1862. doi: 10.3390/rs11161862

[57] Alves KS, Rothmann LA, Del Ponte EM. Linking climate variables to large-scale spatial pattern and risk of citrus huanglongbing: a hierarchical Bayesian modeling approach. Phytopathology. 2022;112: 189–196. doi: 10.1094/PHYTO-05-21-0219-FI 34340530

