# Supplementary material for: Modelling the spread and mitigation of an emerging vector-borne pathogen: Citrus greening in the U.S
Source: PLoS Comput Biol. 2023 Jun 2;19(6):e1010156. doi: 10.1371/journal.pcbi.1010156 (PMC10266658; doi:10.1371/journal.pcbi.1010156)
Supplement: S1 Text — Contains additional details of data, epidemiological models and for methods parameter estimation and model prediction used in the analyses. (DOCX) [file pcbi.1010156.s001.docx]

S1 Text Supporting Information

Modelling the spread and mitigation of an emerging vector-borne pathogen; citrus greening in the U.S.

Viet-Anh Nguyen^1^, David W. Bartels^2^, Christopher A. Gilligan^1*^

^1^Department of Plant Sciences, University of Cambridge, Cambridge, UK

^2^United States Department of Agriculture, Animal and Plant Health Inspection Service, Plant Protection and Quarantine, Fort Collins, Colorado, USA

^*^Corresponding author. Email: cag1@cam.ac.uk

**List of Contents**

[S1 Text Technical Appendix 1](#_Toc127981035)

[Data 1](#_Toc127981036)

[Epidemiological models 2](#_Toc127981037)

[Parameter estimation 6](#_Toc127981038)

[Model prediction 9](#_Toc127981039)

[Data and code availability: 11](#_Toc127981040)

[Supplementary References 11](#_Toc127981041)

## S1 Text Technical Appendix

### Data

##### HLB survey data

The U.S. Department of Agriculture (USDA) has carried out intensive surveys for early detection of HLB in Texas since 2008. In California, the surveys were conducted by the California Department of Food and Agriculture (CDFA). The survey programs collected leaf and psyllid samples on residential citrus trees from the whole citrus landscape in each region. In Texas, samples were also collected in commercial citrus orchards. The survey team collected leaves with HLB-like symptoms and psyllids based on a risk-based survey model [1]. Samples were sent for testing of the Las bacteria to certified laboratories using the approved USDA diagnostic protocol utilizing qPCR technology. For diagnostic tests of leaf tissue samples, a Ct value less than 36 (over 40 cycles) classified the sample as HLB positive. A Ct threshold of 38 was used for samples of vectors. Besides diagnostic samples, we also have access to records of survey visits in which no symptomatic leaves were found. We aggregated the two datasets and considered each survey trial, whether any leaf samples were collected or not, as a data instance. We matched a data instance to a grid cell if its recorded location fell within the containing boundary of the cell. Each data instance was therefore identified by its containing cell and visit date and contained the diagnostic results of the collected leaf samples (if any). We merged cases with the same identification (cell, date) into a single data instance and summed up their diagnostic results into a single diagnostic outcome. A cell was negative at time $t$ if no leaf sample had been collected or all samples were tested negative up to that time, and positive if at least one leaf sample tested positive. Figures 1B and S1B mapped the locations of HLB positives and negatives for vector and plant samples for Texas, respectively. Fig. 1D shows HLB plant positives and negatives in southern California.

Citrus tree density was estimated from commercial citrus survey maps and from county tax parcel information. Both Texas and California maintain updated GIS layers of commercial citrus orchards and the planting rate per acre is fairly standard within each state. The backyard citrus trees were estimated based on HLB survey data in each state. Survey crews would document the number and type of citrus trees present on the parcels visited. We then utilized county parcel data to determine residential properties and applied the estimates of parcels with citrus trees and the number of trees per parcel across each area. As surveys have continued on more properties, the estimate of backyard citrus trees has remained reasonable accurate.

##### ACP trapping data

In Texas, ACP was widespread in the citrus growing region before HLB surveys were started, and trapping was conducted only in localized areas in conjunction with area-wide management research. The presence and spread of ACP in California were compiled through data sets from several trapping networks. Trapping was conducted by CDFA using 14 x 23 cm double-sided yellow sticky panel traps, and ACP were trapped throughout Southern California from 2008 – 2014 [2]. CDFA also trapped ACP in the San Joaquin Valley in Central California on yellow sticky traps run for both the ACP and Glassy-winged Sharpshooter Programs with data available through October 2017. Both of these data sets represent presence-only data rather than counts per trap. Additional data were included from the yellow sticky panel trap network run by the Citrus Pest and Disease Prevention Program in commercial citrus orchards in both Southern California and the San Joaquin Valley. Data were available from 2009 – 2012. We used ACP trapping data between 2012 to 2017 (Fig. 1E) to validate the epidemiological model for ACP spread in the Central Valley and to initialize the joint ACP/HLB model for the region.

### Epidemiological models

A detailed description of the epidemiological models is presented here. Some material from the main text is reproduced where it helps to description and flow.

##### HLB and ACP spread models

When the vector is endemic, we consider four infection categories for each citrus grid cell: Susceptible, Exposed, Infectious, and Detected (Fig. 2A). A cell is susceptible if all the trees in the cell are healthy or free from the Las bacteria. An exposed cell contains infected trees but is not yet infectious to trees in other cells. An infectious cell can transmit Las bacteria to other cells via the movement of ACP. Finally, a cell becomes detected as a survey team collects a positive HLB sample confirmed by a qPCR diagnostic test. The transition of a cell from being infectious to detected requires two steps: first, infectious trees must show symptoms, and second, the site must be visited by a survey team in searching for the symptomatic trees.

We extended the HLB spread model to account for the fact that the underlying ACP population in the Central Valley is still spreading and has not fully invaded the region (Fig. 2B). In addition to the previously defined HLB infection compartments (HLB susceptible, HLB exposed, and HLB infectious), we consider three compartments for ACP infestation status in a grid cell: *S^V^, E^V^ I^V^.* To connect the dynamics of ACP infestation to HLB infection, we introduced a new epidemic category: ACP + HLB infected (*I^V‑P^*), which marks cells that contain HLB infected vectors (Fig. 2B). The presence of HLB-infected vectors in a cell is due to the presence of HLB infectious trees inside the cell, or the arrival of Las-carrying vectors from a nearby cell.

##### Modelling HLB exposure

An ‘HLB susceptible’ cell $i$ is exposed to infection as the first tree in the cell gets infected. The exposure can happen via either primary or secondary transmission. Primary transmission can originate from either the introduction of infected trees and products by trade and other human-mediated movements or from infected vectors arriving from external environments, including across the Mexico border. Since we did not know the quantitative effects that such events have on HLB transmission, we used a parameter $\epsilon$ to represent HLB importation rate by human activities and two parameters $\varepsilon_{W}$ and $\varepsilon_{B}$ to represent the rates of transmission via external vectors for sites in close distance to the Mexico border and sites far away from the border, respectively.

Secondary transmission from an infectious to a susceptible cell is due to the flux of vectors moving between the two cells. The number of vectors moving towards a cell $i$ from cell $j$ depends on two factors: the spatial distance between two cells and the availability of new flushes in the destination cell $i$. We used an exponential isotropic kernel, $K_{\alpha}\left( r \right)\propto e^{-r/\alpha}$ where $\alpha$ represents the dispersal scale, to depict the dependency of movement rate on the spatial distance $r$ between the cells. We established the overall strength of spatial coupling between grid cells by considering a mechanistic model that addresses vector movement and feeding, described below. As vector dynamics occur at faster rates than HLB epidemiological dynamics, the vector counts quickly converge to equilibrium values that we used in calculating the HLB exposure rate as follows.

$$\begin{aligned} \phi_{i}\left( t \right)=\epsilon+\left[ \left( 1-b_{i} \right)\varepsilon_{W}+b_{i}\varepsilon_{B} \right]h_{i}+\beta_{P}h_{i}\sum_{j} {I_{j}^{V}\left( t \right)I}_{j}^{P}\left( t \right)\frac{\kappa_{j}h_{j}K_{\alpha}\left( r_{ij} \right)}{\mu+ \sum_{j^{'}} \kappa_{j^{'}}h_{j^{'}}K_{\alpha}\left( r_{jj^{'}} \right)} \#\left( 1 \right) \end{aligned}$$

Besides the parameters $\epsilon,\varepsilon_{B}, \varepsilon_{W}, \alpha, \beta_{P}, \mu$, the exposure rate incorporated the following data for the susceptible cell $i$ and infectious cell $j$:

- Citrus density $h_{i}$
- Indicator of whether the cell was adjacent to the Mexican border $b_{i}$
- Vector density weight $\kappa_{i}= \kappa_{i}^{C}\kappa_{i}^{W}$, which incorporated the effect of vector control $\kappa_{i}^{C}$ and weather suitability to vector development $\kappa_{i}^{W}$ described below.

As commercial growers applied sprays in November and early February in a coordinated manner, they were able to reduce the density of vector in commercial orchards. We used $\kappa_{i}^{C}$ to represent the relative weight of vector capacity in plantations in comparison with residential trees. We assumed that the vector density in cell $i$ decrease as the proportion of commercial trees in the cell, $f_{i}^{C}$, increases. The parameter $\eta$ denotes the efficiency of vector control measures applied to a cell.

$$\kappa_{i}^{C}=1-\eta f_{i}^{C}$$

Daily temperatures and other weather variables affect vector density by leveraging or slowing down the development of eggs, nymphs, and adult vectors. We used $\kappa_{i}^{W}$ to account for the variation of vector density at different locations due to their corresponding weather patterns. We used the modified Logan function $r(\cdot)$ provided by [3] to calculate the vector development rate in a day given the day’s temperatures, $r_{id}=r(w_{id})$. The function addresses temperatures in the range of 10 to 33 °C and assumes that no vector growth occurs beyond this range. We computed the expected weather-driven vector capacity coefficient $\kappa_{i}^{W}$ by averaging over the development rates for the whole year.

$\kappa_{i}^{W}= \sum_{d=1}^{365} r_{id}/365$

The unknown parameters $\epsilon,\varepsilon_{B}, \varepsilon_{W}, \alpha, \beta_{P}, \mu, \eta$ were estimated from the plant diagnostic data of the Texas HLB survey using a DA-MCMC algorithm under a Bayesian inference framework. We used uninformative prior distributions for all parameters.

##### Modelling vector flux between cells

Here we develop a mechanistic model for between-cell vector fluxes, which drive secondary transmission in HLB and ACP epidemiological models. The model constructs the equilibrium abundance of psyllids moving to and feeding in a citrus grid cell. In doing so, it considers the rates of dispersal and also the birth and death rates of vectors.

At an arbitrary time $t,$ we distinguish vectors by their behaviours: $V_{i}$ denotes the number of vectors feeding in cell $i$, and $V_{j\to i}$ denotes the number of vectors moving from cell $j$ to feed on a flush in cell $i$. We let $\delta$ be the rate at which a moving vector arrives in and feeds on a tree in the destination cell, and model the rate at which a vector in cell $j$ finishes feeding and moves to another cell $i$ by the dispersal rate

$$\rho_{j\to i}=\overset{\sim}{\rho}\kappa_{i}h_{i}K_{\alpha}(r_{ij})$$

where $K_{\alpha}(\cdot)$ is an isotropic dispersal kernel parameterised by a dispersal scale $\alpha$, and $\tilde{\rho}$ is a normalisation constant. The movement rate to cell $i$ is proportional to the number of trees $h_{i}$ and vector capacity ratio $\kappa_{i}$ - reflecting the flushes-searching behaviour of vectors and the chance of successful feeding in the destination cell, respectively.

The dynamics for $V_{i}$ and $V_{j\to i}$, for $i, j\in\{1, \ldots, L\}$ with $L$ being the number of citrus cells under consideration, are given by:

$\frac{\partial V_{i}}{\partial t}=b_{v}V_{i}+\delta\sum_{j\neq i} V_{j\to i}-V_{i}\sum_{j\neq i} \rho_{i\to j}-d_{v}V_{i}$,

$\frac{\partial V_{j\to i}}{\partial t}=V_{j}\rho_{j\to i}-\delta V_{j\to i}-d_{v}V_{j\to i}$,

where $b_{v}$ and $d_{v}$ denote vector birth and death rates.

We consider the situation that the modelling region has been fully infested with ACP before introducing CLas, which is the case for Texas. Each cell $i$ is associated with a carrying capacity ${\overset{\sim}{\kappa}}_{i}=\tilde{\kappa}\kappa_{i}$where $\tilde{\kappa}$ is the maximal load of vectors a citrus tree could sustain through flushing and the fraction $0< \kappa_{i}< 1$ allows for capacity reduction due to natural or human intervention. This puts a constraint on the total number of vectors that successfully feed in cell $i$, i.e. $V_{i}(t)+\sum_{j\neq i} V_{j\to i}(t)={\overset{\sim}{\kappa}}_{i}h_{i}$, where again $h_{i}$ denotes the number of trees in the cell. Plugging this constraint into the dynamic equations for $V_{i}$ and $V_{j\to i}$, we arrive at closed-form solutions to the variables:

$V_{i}(t)=\frac{\delta{\overset{\sim}{\kappa}}_{i}h_{i}}{c_{i}}+e^{-c_{i}t}(V_{i}(0)-\frac{\delta{\overset{\sim}{\kappa}}_{i}h_{i}}{c_{i}})$, with$\text{ }c_{i}=\sum_{j\neq i} \rho_{i\to j}+\delta+d_{v}-b_{v},$

$V_{j\to i}(t)=\frac{\delta{\overset{\sim}{\kappa}}_{j}h_{j}\rho_{j\to i}}{c_{j}c_{0}}+e^{-c_{0}t}(N_{j\to i}(0)-\frac{\delta{\overset{\sim}{\kappa}}_{j}h_{j}\rho_{j\to i}}{c_{j}c_{0}})+(e^{-c_{j}t}-e^{-(c_{j}+c_{0})t})(V_{i}(0)-\frac{\delta{\overset{\sim}{\kappa}}_{i}h_{i}}{c_{j}})\frac{\rho_{j\to i}}{c_{0}}$,

with $c_{0}=\delta+d_{v}$.

The relaxation to equilibrium is driven by the characteristic rates $c_{i}$ and $c_{0}$ in the exponentials. Since $b_{v},d_{v}\ll\delta$, we can ignore the contribution of birth and death rates. We expect vectors to move and feed multiple times per day, hence $\left\{ c_{i} \right\}_{i = 1\ldots L}$ and $c_{0}$ are significantly larger than the rates at which epidemiological transitions occur at the cell level. $V_{i}$ and $V_{j\to i}$ therefore quickly converge to the following equilibrium values with the dispersal rate plugged in:

$V_{i}(t)=\frac{\delta{\overset{\sim}{\kappa}}_{i}h_{i}}{\rho_{i}+\mu}$ and $V_{j\to i}(t)=\frac{\delta\overset{\sim}{\kappa}\overset{\sim}{\rho}\kappa_{j}h_{j}\kappa_{i}h_{i}K_{\alpha}(r_{ij})}{(\rho_{j}+\mu)c_{0}}$,

where $\mu=(\delta+d_{v}-b_{v})/\overset{\sim}{\rho}\approx\delta/\overset{\sim}{\rho}$ as $b_{v},d_{v}\ll\delta$, and $\rho_{i}=\sum_{i^{'}\neq i} \kappa_{i^{'}}h_{i^{'}}K_{\alpha}(r_{ii^{'}})$. We can use these steady states for subsequent calculations of the epidemiological transition rates.

Let $X_{i}, Y_{i}, Z_{i}$ represent the number of susceptible, exposed, and infectious trees in cell $i$ at time $t$, the numbers of infectious vectors feeding in and moving from cell $i$ are approximately $V_{i}^{+}=\tau_{v}V_{i}I_{i}$ and $V_{i\to j}^{+}=\tau_{v}V_{i\to j}I_{i}$, where $I_{i} = Z_{i}/h_{i}$ is the proportion of infectious trees in cell $i$, and $\tau_{v}$ is the tree-to-vector transmission efficacy. Disease epidemiological dynamics in relation to the feeding and moving patterns of vectors are given by

$\frac{\partial X_{i}}{\partial t}=b_{h}X_{i}-\frac{\tau_{h}X_{i}}{v_{i}}(V_{i}^{+}+\delta\sum_{j\neq i} V_{j\to i}^{+})-d_{h}X_{i}$,

$\frac{\partial Y_{i}}{\partial t}=\frac{\tau_{h}X_{i}}{v_{i}}(V_{i}^{+}+\delta\sum_{j\neq i} V_{j\to i}^{+})-\gamma Y_{i}-d_{h}Y_{i}$,

$\frac{\partial Z_{i}}{\partial t}=\gamma Y_{i}-d_{h}Z_{i}$.

where $\tau_{h}$ is the vector-to-tree transmission rate, $v_{i}$ is the total number of vectors feeding in cell $i$ at time $t$, $\gamma$ represents the rate at which exposed trees progress to being infectious, and $b_{h}, d_{h}$ denote tree birth and death rates. Here we consider only secondary infection facilitated by vectors moving among trees within the modelling region. The infection process comprises two sources: within-cell bulking up due to movement of local vectors $V_{i}^{+}$ and between-cell transmission due to the arrival of vectors from other cells $V_{j\to i}^{+}$.

Under the meta-population setting, a susceptible cell is exposed to infection when an arbitrary tree in the cell gets exposed to CLas-carrying psyllids feeding. The probability that cell $i$ is exposed to infection is equivalent to $P(Y_{i}(t)=0,Y_{i}(t+\delta t)=1)$ for a vanishingly short period $\delta t$. We can therefore construct the infection rate on a susceptible cell $i$ at a time $t$ using the above results as follows:

$\phi_{i}^{\text{secondary}}(t)=\frac{\tau_{h}h_{i}}{v_{i}}\cdot\delta\sum_{j\neq i} V_{j\to i}^{+}=\frac{\tau_{h}\tau_{v}\delta}{{\overset{\sim}{\kappa}}_{i}}\sum_{j\neq i} V_{j\to i}I_{j}=\beta h_{i}\sum_{j\neq i} \frac{\kappa_{j}h_{j}K_{\alpha}(r_{ij})I_{j}}{\rho_{j}+\mu}$,

where $\beta=\frac{\tau_{h}\tau_{v}\delta^{2}\overset{\sim}{\rho}}{c_{0}}$ and $\rho_{j}=\sum_{j^{'}\neq j} \kappa_{j^{'}}h_{j^{'}}K_{\alpha}(r_{jj^{'}})$.

##### Modelling ACP exposure

An ‘ACP susceptible’ cell $i$ is exposed to an infestation when the first few vectors arrive in the cell either from nearby sites or transported from external environments. We refer to these mechanisms as secondary and primary infestation and used parameters $\beta_{V}$ and $\varepsilon_{V}$, respectively, to represent the rates of infestation. We calculated the force of ACP exposure to a susceptible cell $i$ analogously to the pressure of HLB exposure (Eq. 1) as follows:

$$\begin{aligned} \psi_{i}\left( t \right)=\varepsilon_{V}h_{i}+ \beta_{V}h_{i}\sum_{j} I_{j}^{V}\left( t \right)\frac{\kappa_{j}h_{j}K_{\alpha}\left( r_{ij} \right)}{\mu+ \sum_{j^{'}} \kappa_{j^{'}}h_{j^{'}}K_{\alpha}\left( r_{jj^{'}} \right)} \#\left( 2 \right) \end{aligned}$$

Since we used the ACP spread model for the Central Valley only, we can discard the extra risk of primary infection along the Mexico border.

As we cannot estimate the unknown parameters $\varepsilon_{V}, \beta_{V}$ directly from California ACP trapping data, we assumed that $\varepsilon_{V}\approx\varepsilon_{W}$ and estimated $\beta_{V}$ from the Texas HLB survey data using the ‘ACP Infested’-to-‘ACP + HLB Infected’ model described below.

##### Modelling the transition from ‘ACP Infested’ to ‘ACP + HLB Infected’

An ‘ACP infested’ cell transitions to ‘ACP + HLB Infected’ cell when vectors in cell $i$ acquire CLas bacteria either by feeding on infectious trees inside the cell or by migrating from a nearby infectious cell. The former is also known as the bulking up of infected vectors inside the cell and is driven by an unknown parameter $\xi$. The rate at which infected vectors migrate from an ‘ACP + HLB Infected’ cell to an ‘ACP Infested’ site is equivalent to the rate $\beta_{V}$ at which vectors from an ‘ACP Infested’ cell arrive in an ‘ACP Susceptible’ cell under the assumption that CLas-carrying vectors behave similarly to uninfected vectors. Using the same dynamic model of vector movement and feeding as before, we can calculate the transition force as follows:

$$\begin{aligned} \varphi_{i}\left( t \right)=S_{i}^{P}\left( t \right)h_{i}\left( \xi\frac{I_{i}^{V}\left( t \right)E_{i}^{P}\left( t \right)}{\mu+ \sum_{i^{'}\neq i} \kappa_{i^{'}}h_{i^{'}}K_{\alpha}\left( r_{ii^{'}} \right)}+ \beta_{V}\sum_{j\neq i} I_{j}^{V}\left( t \right)I_{j}^{P}\left( t \right)\frac{\kappa_{j}h_{j}K_{\alpha}\left( r_{ij} \right)}{\mu+ \sum_{j^{'}\neq j} \kappa_{j^{'}}h_{j^{'}}K_{\alpha}\left( r_{jj^{'}} \right)} \right) \#\left( 3 \right) \end{aligned}$$

The unknown parameters $\xi, \beta_{V}$ were estimated using the vector diagnostic data from the Texas HLB survey. Since we carried out parameter estimation under a Bayesian inference framework, we can use the previously acquired posterior estimates for $\alpha,\mu,\eta$to complement the sparsity of vector diagnostic data.

##### Latent period parameters

The time it takes for a cell to transition from ‘HLB Exposed’ to ‘HLB Infectious’ is known as the HLB latent period. It starts when a tree gets exposed to HLB infection and ends as a significant number of trees in the cell became infectious so that the amount of Las-carrying vectors that move away from the cell is large enough to cause infection in another cell. We followed [4] to use a seasonally-forced model for the rate of infectiousness onset.

$$\begin{aligned} \gamma\left( t \right)=2a_{P}\sin^{2} \frac{\pi t}{365} \#\left( 4 \right) \end{aligned}$$

$a_{P}$ denotes the average rate at which a cell moves from ‘HLB Exposed’ to ‘HLB Infectious’. We set the parameter to empirical estimates from in-orchard and in-nursery observations for trees more than ten years of age, which reported an average latent period of 15 months, i.e. $a_{P}=0.8$. We assumed that the specific latent period for each cell follows an exponential distribution with rate $\gamma\left( t \right)$. As such, the latent periods vary among cells and include very short durations due to the exponential form of the model.

Analogously, the ACP latent period indicates the time it takes for a cell to transition from ‘ACP Exposed’ to ‘ACP Infested’. We used the same seasonally-forced model as the model for HLB latent period described above, with $a_{P}$ replaced by $a_{V}=0.042$. The rate is equivalent to an expected ACP latent period of 15 days, which covers the duration for nymphs to develop into adult psyllids.

### Parameter estimation

##### Bayesian inference

We adopt a Bayesian approach to estimate the epidemiological parameters $\Theta$ from noisy survey data $Y$. Since the epidemic trajectory $X$ or the times at which epidemiological transitions occurred at each cell were unobservable, we treated them as unknown random variables and integrated them using a data-augmented MCMC algorithm [5,6]. The Bayes rule provided a way to compute the joint posterior distribution of parameters and epidemic trajectory from the parameter prior $P(\Theta)$, model likelihood $P(X|\Theta)$, and data likelihood $P(Y|X, \Theta)$

$$P\left( \Theta, X | Y \right)\propto P(\Theta)P(X|\Theta)P(Y|X, \Theta)$$

An MCMC algorithm approximates the posterior distribution by constructing a Markov chain that has the desired distribution as its equilibrium distribution. Samples generated from the chain after a burn-in period form a representation of the posterior. In each iteration, the algorithm alternatingly draws samples of the parameters $\Theta$ and epidemic trajectory $X$. We used the Metropolis-Hasting method [7] to construct samplers for both model parameters and components of the unobservable epidemic trajectory. We used simple log-normal proposal distributions for $\epsilon, \varepsilon_{W}, \varepsilon_{B},\alpha,\mu, \eta, \xi$, and Gibbs samplers for $\beta_{P},\beta_{V}$. We used the randomised construction [8] of Markov trajectory and exact inference algorithms for hidden Markov models [9] to construct improved samplers for updating transition times. Thanks to considering an epidemic trajectory as a whole [10], these improvements led to faster mixing than the usual adaptation of the reversible jump method for epidemiological inference.

##### Likelihoods for estimating parameters of HLB exposure model

We used the stochastic construction of the HLB spread model to compute the model likelihood or the probability of an epidemic trajectory $X$ conditioned on a set of parameter values $\Theta$. An epidemic trajectory for a cell $i$ over the modelling period ${[t}_{0},t_{F}]$ comprises the initial epidemic state $x_{0}^{i}$ and the times $t_{E}^{i}, t_{I}^{i}$ at which the cell transitions from ‘HLB Susceptible’ to ‘HLB Exposed’ and ‘HLB Exposed’ to ‘HLB Infectious’ respectively. Let $\mathcal{X}_{ab}$ be the set of cell indices that were in state $a$ at time $t_{0}$ and state $b$ at time $t_{F}$, e.g. $\mathcal{X}_{SS}$ indicates cells that were never exposed to infection, and $\mathcal{X}_{SE}$ represents cells that were exposed but never infectious. The model likelihood is given as follows:

$$P\left( X | \Theta\right)= \prod_{i} P(x_{0}^{i})\times\prod_{i\in\mathcal{X}_{SS}} e^{-\Phi_{i}(t_{0},t_{F})}\times\prod_{i\in\mathcal{X}_{EE}} e^{-\Gamma(t_{0},t_{F})}\times\prod_{i\in\mathcal{X}_{SE}} {\phi_{i}(t_{E}^{i})e}^{-\Phi_{i}(t_{0},t_{E}^{i})}e^{-\Gamma(t_{E}^{i}, t_{F})}\times\prod_{i\in\mathcal{X}_{EI}} \gamma{(t_{I}^{i})e}^{-\Gamma(t_{0},t_{I}^{i})}\times\prod_{i\in\mathcal{X}_{SI}} {\phi_{i}\left( t_{E}^{i} \right)e}^{-\Phi_{i\left( t_{0},t_{F} \right)}}\gamma\left( t_{I}^{i} \right)e^{-\Gamma\left( t_{E}^{i}, t_{I}^{i} \right)} \left( 5 \right)$$

$\phi_{i}\left( t \right)$and $\gamma(t)$ are defined in equations (1) and (4), and $\Phi_{i}\left( t \right)\left( a, b \right)=\int_{a}^{b} \phi_{i}(t)dt$ and $\Gamma\left( a, b \right)=\int_{a}^{b} \gamma\left( t \right)dt$ compute the cumulative rates in the period $[a, b)$. We used a uniform prior distribution for the initial epidemic state, i.e. $P\left( x_{0}^{i} \right)\propto1$.

We used probabilistic observation models to relate an epidemic trajectory to noisy diagnostic data from the Texas state-wide HLB survey. Observed data $y^{i}$ collected from cell $i$ comprise a set of trials, each associated with the date of the visit and the diagnostic outcome of that date. We assumed observational independence among cells given the knowledge of the hidden epidemiological events, i.e. $P\left( Y | X, \Theta\right)= \prod_{i} P\left( y^{i} | x^{i}, \Theta\right).$ In doing so, we attributed temporal and spatial correlations in the observed data to the underlying epidemic and not biases in surveying procedures. A survey trial has two possible outcomes: positive (at least one HLB positive leaf sample was collected), and negative (no symptomatic leaf was found, or all collected samples were HLB negative). Let $y_{P+}^{i}$ and $y_{P-}^{i}$ be the set of times of positive and negative trials, and $t_{P+}^{i}$ be the time at which the first positive plant sample was collected. We used two alternative observation models to relate observed data to the time of infectiousness onset, $t_{I}^{i}$, in each cell:

$$\begin{aligned} P\left( y^{i} | x^{i}, \pi\right)= \prod_{t\in y_{P+}^{i}} \pi\mathbf{1}\left( t>t_{I}^{i} \right)\times\prod_{t\in y_{P-}^{i}} \left( 1-\pi\mathbf{1}\left( t>t_{I}^{i} \right) \right) \#\left( 6 \right) \end{aligned}$$

$$\begin{aligned} P\left( y^{i} | x^{i}, \sigma\right)=\sigma e^{-\sigma\left( t_{P+}^{i}-t_{I}^{i} \right)}\#\left( 7 \right) \end{aligned}$$

Each model aims to uncover one of the two parameters representing the data collection process. The parameter $\pi$ represents the probability of a positive sample to be collected from an infectious cell. Note that this task is not trivial due to the potentially large number of trees in a cell and the similarity in symptoms of HLB infected and under-nutritional leaves. The parameter $\sigma$ indicates the expected duration from becoming infectious to getting detected, which depends on not only how thoroughly a cell was checked during each visit but also how often a team returned to a negative cell. We used the former model during parameter estimation, and the latter model in predicting the expected patterns of disease detection in the future.

##### Likelihoods for estimating parameters of ACP exposure model

To estimate the rate for secondary ACP infestation $\beta_{V}$, we augmented the epidemic trajectory in each cell $i$ to include the time $t_{V}^{i}$ at which HLB infected vectors arrived in the cell. Let $\mathcal{X}_{V+}$ and $\mathcal{X}_{V-}$ denote the set of cell indices with and without HLB positive vectors at the end time $t_{F}$ respectively. The model likelihood is therefore given analogously to Eq. 5 as follows:

$$\begin{aligned} P\left( X | \Theta\right)= \prod_{i} P\left( x_{0}^{i} \right)\times\prod_{i\in\mathcal{X}_{SS}} e^{-\Phi_{i}\left( t_{0},t_{F} \right)}\times\prod_{i\in\mathcal{X}_{EE}} e^{-\Gamma\left( t_{0},t_{F} \right)}\times\prod_{i\in\mathcal{X}_{SE}} {\phi_{i}\left( t_{E}^{i} \right)e}^{-\Phi_{i}\left( t_{0},t_{E}^{i} \right)}e^{-\Gamma\left( t_{E}^{i}, t_{F} \right)}\times\\ \prod_{i\in\mathcal{X}_{EI}} \gamma{\left( t_{I}^{i} \right)e}^{-\Gamma\left( t_{0},t_{I}^{i} \right)}\times\prod_{i\in\mathcal{X}_{SI}} {\phi_{i}\left( t_{E}^{i} \right)e}^{-\Phi_{i\left( t_{0},t_{F} \right)}}\gamma\left( t_{I}^{i} \right)e^{-\Gamma\left( t_{E}^{i}, t_{I}^{i} \right)}\times\\ \prod_{i\in\mathcal{X}_{V+}} \varphi_{i}\left( t_{V}^{i} \right)e^{-\Psi_{i}\left( t_{0},t_{V}^{i} \right)} \times\prod_{i\in\mathcal{X}_{V-}} e^{-\Psi_{i}\left( t_{0},t_{F} \right)} \#\left( 8 \right) \end{aligned}$$

$\Psi_{i}(t)=\int_{a}^{b} \varphi_{i}\left( t \right)dt$ computes the cumulative pressure in the period $[a, b)$.

We also extended the observed diagnostic data to include both plant and vector samples from the HLB survey in Texas. Besides the set of positive and negative trials for plant samples $y_{P+}^{i}$ and $y_{P-}^{i}$, we also had a set of positive and negative trials for vector samples $y_{V+}^{i}$ and $y_{V-}^{i}$. The data likelihood can be written analogously to Eq. 6 as follows:

$$P\left( y^{i} | x^{i}, \pi_{P}, \pi_{V} \right)= \prod_{t\in y_{P+}^{i}} \pi_{P}\mathbf{1}\left( t>t_{I}^{i} \right)\times\prod_{t\in y_{P-}^{i}} \left( 1-\pi_{P}\mathbf{1}\left( t>t_{I}^{i} \right) \right)\times$$

$$\begin{aligned} \prod_{t\in y_{V+}^{i}} \pi_{V}\mathbf{1}\left( t>t_{V}^{i} \right)\times\prod_{t\in y_{V-}^{i}} \left( 1-\pi_{V}\mathbf{1}\left( t>t_{V}^{i} \right) \right) \#\left( 9 \right) \end{aligned}$$

$\pi_{P}$ and $\pi_{V}$ represent the respective probabilities of collecting HLB-positive plant and vector samples from an infectious site. $\pi_{P}$ is equivalent to the collection probability $\pi$ estimated for the HLB exposure model, and $\pi_{V}$ is treated as an auxiliary parameter for which the DA-MCMC algorithm must account.

##### DA-MCMC algorithms for parameter estimation

To estimate the posterior distribution of the parameters $\Theta_{HLB}=(\epsilon,\varepsilon_{B}, \varepsilon_{W}, \alpha, \beta_{P}, \mu, \eta, \pi, \sigma)$ for the HLB exposure model, we used a data augmented Markov chain Monte Carlo (DA-MCMC) algorithm with Metropolis-Hasting (MH) samplers. We expanded the parameter space to include the unobservable epidemic trajectory $X_{HLB}=\left\{ x_{0}^{i}, t_{E}^{i}, t_{I}^{i} \right\}_{i}$ and sampled iteratively from the joint parameter-trajectory space $\Theta_{HLB}\times X_{HLB}$. The $(n+1)$th iteration uses sample values $(\Theta^{\left( n \right)}, X^{\left( n \right)})$ from the previous iteration and generates updated values as follows:

1. For each parameter $\theta\in\Theta_{HLB}$, we proposed a new value $\hat{\theta}\sim Q({\hat{\theta}|\theta}^{\left( n \right)}, \tau)$, where $\tau$ is the step size of the proposal distribution $Q$ and can be adjusted to improve convergence. The iteration accepts the new value $\hat{\theta}$ with probability $\min\{1,P(X^{\left( n \right)}, Y,\hat{\Theta})Q(\Theta^{\left( n \right)}|\hat{\Theta},\tau)/P(X^{\left( n \right)}, Y, \Theta^{\left( n \right)})Q(\hat{\Theta}|\Theta^{\left( n \right)}, \tau)\}$ where $\hat{\Theta}$ is the set of parameter values from the previous iteration with $\theta^{\left( n \right)}$ replaced by $\hat{\theta}$ and $P(X, Y, \Theta)=P(X|\Theta)P(Y|X)P(\Theta)$. The model and data likelihoods are given in Eq. (5-7), and we used uninformative priors for all parameters. We adopted $\hat{\Theta}$ for the $\left( n+1 \right)th$ parameter samples if $\hat{\theta}$ is accepted and reused $\Theta^{\left( n \right)}$ if the new value is rejected. We used log-normal densities as the proposal distribution for parameters $\epsilon,\varepsilon_{B}, \varepsilon_{W}, \alpha, \mu, \eta, \pi$, and conditional densities for $\beta_{P}$ and $\sigma$. Using conditional densities to propose parameter values led to acceptance probabilities of 1, and is also known as the Gibbs update.
2. For each trajectory time/state, e.g. $t_{E}^{i}\in X$, we generated a new value for the variable by using the randomised construction of a Markov trajectory. An event $t_{E}^{i}$ is equivalent to a discrete-time Markov chain $Z_{U}=\{(u_{m}, z_{m})\}$ where $u_{m}\in[t_{0},t_{F}]$ denotes the $m$^th^ time index of the chain, and $z_{m}\in\{S, E\}$ indicates the chain state at the corresponding time. The time at which $z_{m}$ switches from S to E (i.e. $z_{m-1}=S, z_{m}=E$) represents the exposure time of the cell. Given the event value from the previous iteration $t^{\left( n \right)}$, we generated a new event value as follows:
   1. Draw two new sets of index times $\{u_{1},\ldots, u_{m}\}$ and $\{u_{m+1}, \ldots, u_{M}\}$ from two inhomogeneous Poisson processes with intensity functions $\omega\phi_{i}\left( t \right)$, $t\in(t_{0},t^{\left( n \right)})$ and $\left( \omega-1 \right)\phi_{i}\left( t \right),$ $t\in\left( t^{\left( n \right)}, t_{F} \right)$ respectively. The coefficient $\omega>1$ drives the resolution of the sampled index times and can be adjusted to improve convergence. Function $\phi_{i}(t)$ (Eq. 1) represents the rate of exposure to HLB infection. (To draw index times for $t_{I}^{i}$, we replaced $\phi_{i}\left( t \right)$ with $\gamma_{i}(t)$.) We joined the two new sets with the current event value to form the complete potential transition times $\boldsymbol{u}=\left\{ u_{1},\ldots, u_{m}, t^{\left( n \right)}, u_{m+1}, \ldots, u_{M} \right\}.$
   2. Infer the set of states $\boldsymbol{z}=\{z_{m}\}$ corresponding to the proposed transition times $\boldsymbol{u}$ by sampling from the conditional posterior distribution $P(\boldsymbol{z}|X^{\left( n \right)}, \Theta^{\left( n \right)})$. Given $\boldsymbol{u}$, $\boldsymbol{z}$ is equivalent to a hidden Markov chain for which $X^{\left( n \right)}$ constitutes a sequence of noisy observations of the hidden states. We can, therefore, use the forwards-backwards algorithm to directly infer $\boldsymbol{z.}$ Note that due to the spatial couplings among cells, we treated $X^{\left( n \right)}$ not only as observations but also use the data to compute the transition probabilities of the chain.
   3. We can easily decipher the new event value given $(\boldsymbol{u, z})$. Note that here we accepted all new event values as the acceptance probability always equalled 1.

To estimate the ACP invasion rate parameter $\beta_{V}$ from Texas HLB survey data, we further extended the parameter space to include the extra parameters, i.e. $\Theta_{ACP}=\Theta_{HLB}\times(\beta_{V}, \xi, \pi_{V})$, and the epidemic trajectory space to include times of HLB infected vectors arriving in a cell, i.e. $X_{ACP}=\{x_{0}^{i},t_{V}^{i}, t_{E}^{i}, t_{I}^{i}\}$. We used a DA-MCMC algorithm to draw posterior samples from the joint parameter-trajectory space $\Theta_{ACP}\times X_{ACP}$. The $(n+1)$^th^ iteration uses sample values $(\Theta^{\left( n \right)}, X^{\left( n \right)})$ from the previous iteration and generates updated values as follows:

1. For each parameter ${\theta\in(\beta}_{V},\xi, \pi_{V})$, we sampled a new value $\theta^{\left( n+1 \right)}$ using an independent MH sample as before. We used uninformative priors for the new parameters and computed the acceptance probability using the likelihoods given in Eq. (8-9). For a parameter $\theta'\in\Theta_{HLB}$, we drew values randomly from the set of posterior samples obtained in MCMC chains for HLB parameter estimation.
2. We generated a new value for each of the trajectory time/state variable in $X_{ACP}$ using inhomogeneous Poisson processes and the forwards-backwards algorithm as before.

##### Inference of locations of HLB cryptic cells using observed survey data

To infer the locations of HLB Exposed and Infectious cells up to an observation time $T_{obs}$, we used an MCMC simulator analogous to the DA-MCMC algorithms used for parameter estimation. We sampled sets of parameter values from the estimated joint posterior distribution. We simulated the transition time indices $\boldsymbol{u}$ using inhomogeneous Poisson processes as above for the period $\left[ t_{0},T_{obs} \right)$ and inferred the corresponding states $\boldsymbol{z}$ conditioned on the survey data up to time $T_{obs}$ using the HMM forwards-backwards algorithm. We recorded one in every five epidemic trajectory realizations to lessen Markov dependency between simulations.

##### Model validation

**We validated the HLB spread model for Texas** against the Texas HLB survey data collected between Dec 2011 and Oct 2018. We divided the data into a training set and a testing set. The training data comprised diagnostic plant samples collected between December 2011 and August 2016, and the testing data covered samples collected from September 2016 to October 2018. We estimated HLB parameters $\Theta_{HLB}$ using the DA-MCMC algorithm described above from the training data. Given the posterior samples of parameters and initial epidemic states, we randomly drew 1000 sets and simulated epidemics from December 2011 to October 2018 using the Gillespie construction. We then compared the temporal and spatial structure of model predictions with those of the observed survey data. For each epidemic outcome, we considered the prevalence of each HLB infection category as it changed over time and the spatial autocorrelation of all detected trees up to October 2018. We calculated the prevalence by approximating the counts of infected trees in the whole landscape. We used Moran’s I statistic to measure the spatial autocorrelation of HLB infection and ACP infestation as predicted by models and observed in the survey data. The autocorrelation score for an epidemic outcome is given by:

$$I(r_{1},r_{2})=\frac{N}{W}\frac{\sum_{i} \sum_{j} w_{ij}(r_{1},r_{2})\left( x_{i}-\bar{x} \right)\left( x_{j}-\bar{x} \right)}{\sum_{i} \left( x_{i}-\bar{x} \right)^{2}}$$

where $N$ is the number of cells in the landscape, $x_{i}$ is the infection/infestation in cell $i$, $w_{ij}\left( r_{1},r_{2} \right)=1$ if the spatial distance $r_{ij}$ between cells $i$ and $j$ is longer than $r_{1}$ but shorter than $r_{2}$.

**We validated the HLB spread model for southern California** against the California HLB survey data collected between June 2015 and June 2019. We created a training set using samples collected before July 2017 and a testing set using data collected afterwards. We set $T_{obs}$ at this dividing time point and inferred the locations of exposed and infectious cells using the MCMC simulator described above and the training data and the parameter values previously estimated from Texas data. We then generated Gillespie simulations of the HLB epidemics in southern California from $T_{obs}$ to June 2019 using the inferred infected locations and random samples of the parameter posterior. Similar to the validation for Texas, we compared the temporal prevalence and spatial autocorrelation structure of model predictions with those of the observed survey data.

**To validate the ACP spread model for Central Valley,** we used the DA-MCMC algorithm to estimate $\Theta_{ACP}$ from the plant and vector diagnostic data collected in Texas as described above. We then plugged parameter estimates in the ACP spread model for Central Valley and ran Gillespie simulations to reproduce the historic ACP spread in 2015 and 2016. We validated model predictions against the ACP trapping data, which were collected independently from the HLB survey and not used for parameter fitting. We used temporal prevalence as the evaluation metric and compared that of model predictions to the observed trapping data.

### Model prediction

##### Spatiotemporal predictions of HLB spread

To make spatiotemporal predictions of further HLB spread in Texas and southern California until a future time $T_{pred}$, we simulated 1,000 epidemic outcomes and calculated the probabilities of infection in each site at the designated time by averaging over the realizations. We started Texas simulations in December 2011 and southern California simulations in June 2017 and ran simulations more than two years past the final observation time for each region. For each realization, we set model parameters and initial epidemic states with a set of values randomly drawn from their joint posterior distribution. We incorporated the effect of the annual coordinated spraying program to commercial orchards in Texas using the estimated relative control efficiency coefficient $\eta$.

The first citrus leaves tested positive with HLB in southern California were collected in Los Angeles County in March 2012. However, the strain of HLB found at that location does not appear to have spread [11], and we utilized the June 2015 detection as the initiation of the spread. The pathogen has invaded the nearby Orange and Riverside Counties since then (Fig. 1D). We assumed the same effectiveness of coordinated spraying. We then extended the simulator to allow for two additional reactive control measures applied to newly detected locations and their neighbouring area. In particular, California regulators removed HLB infected trees upon positive diagnostic confirmation and put the area surrounding each identified tree under quarantine. The quarantine area evolved as the epidemic progressed and was formed by circles of five-mile radius centring around detected trees. Movement of citrus tree products from the quarantine area to other places were prohibited. We approximated the effect of tree removals in an infectious cell by reducing its infectiousness by a proportion $\pi$ once the cell got detected as HLB positive. $\pi$ denotes the probability of detecting an HLB positive tree in an infectious cell and therefore can be used as a proxy for the proportion of infectiousness identified in a cell. To model the effect that human movement restriction had on HLB transmission, we reduced the infection rate from quarantine cells to other cells by 10%. Simulations using 5% and 20% rate reduction resulted in similar spreading patterns. Although we did not use the data after the starting times to constrain the actual detections observed in the survey, we found a good agreement of model predictions with the testing data for both Texas and southern California.

To jointly predict the potential spread of ACP and HLB in Central Valley, we used the extended HLB spread model in which we conditioned the dynamics of HLB spread on those of ACP spread. HLB infection can only happen between cells fully infested with ACP. We used the ACP trapping data collected from 2017 to 2019 in the area to initialize ACP infestation. Although trapping data were also available for 2012–2016, we did not use them due to the discontinuity in epidemic growth observed between 2016 and 2017. A potential explanation for the epidemic disruption is the 2016 sweltering summer during which the whole citrus region in California had at least ten days with temperatures above 37.8 °C [12]. Vectors were less likely to move out but stay hiding in the cooler parts of the citrus canopy, making it easier for growers to eradicate them. We used locations with inconclusive psyllid samples (Ct values < 38) to set up potential HLB infected sites. Besides the annual dormant spraying of ACP by commercial growers in Central Valley, CDFA teams applied pesticide treatment to citrus trees within a 400m-radius circle surrounding a detected location. If the treated circle intersects with a commercial orchard, ACP control will apply to the whole plantation. We modelled the effect of this ACP reactive treatment program by reducing the infectiousness of a treated cell by the area of the geometric intersection of the boundaries of the treated circle or commercial orchard with those of the cell under consideration. We started the simulations in December 2019 and ran 1,000 simulations for ten years into the future until January 2030. We averaged over simulation outcomes to estimate the ACP infestation and HLB infection probabilities.

##### Evaluation of putative control strategies

To evaluate the impact of a control strategy on the epidemic outcome, we set the corresponding parameter for a selected control treatment to an appropriate value for the strategy to be tested. We then estimated the effect of the implementation by collating over epidemic outcomes generated via Gillespie simulations. To quantify the role of the annual coordinated spraying program in slowing down HLB spread in Texas, we set the control efficiency coefficient $\eta$ to two other alternative values (20% and 50%) besides the value estimated from Texas HLB survey data (80%). We ran 1,000 simulations for the period December 2011 and December 2019 using the previously acquired posterior samples for model parameters and initially infected sites, except for the efficiency of the annual coordinated ACP spraying in commercial orchards, $\eta$. We assumed control efficiency to remain constant throughout the simulation period. We considered the total exposed area at a time $t$ for each simulation by counting the number of 1km^2^ cells in the landscape that had been exposed to HLB infection up to that time. We derived 50%, 75%, and 95% credible intervals using the simulated data samples.

To assess the effect of increasing the radius of quarantine circles to decreasing HLB infectious area in southern California, we used the counts of 1km^2^ cells that had been HLB infectious up to December 2021 as the evaluation metric. We started simulations in June 2017 using the inferred infected locations from data up to this point. We did not make use of either survey data or the quarantine boundaries data available after this time. We evolved the boundaries of the quarantine area as the model predicted new detections. The model used the detection rate estimated from Texas. Besides the implementation of HLB quarantines, we also incorporated the effect of the annual coordinated spraying and the removal of HLB infected trees upon positive diagnosis confirmations. We simulated 1,000 epidemic trajectories for each quarantine radius of 1, 2, 3, …, 14 km and derived the median and credible intervals from the generated samples.

Two parameters that shape the reactive ACP eradication program in Central Valley are the eradication efficiency and the radius of the treated circle centring around an ACP positive site. We measured the effect of changing the parameter values to the infestation area by starting simulations in January 2020 and consider the epidemic outcome in December 2021. We varied the treatment efficiency between 0% and 100% and the eradication radius from 0.1 to 2.0 km. The boundaries of the area under treatment evolved as the model predicted new ACP detections. We assumed an average detection lag of one month, which was the regularity for checking ACP traps by CDFA teams. If the newly constructed treatment circle intersects with the boundary of a commercial grove, we merged the two geometric objects to build an extended treatment area. We computed the area fraction under treatment for each grid cell by considering the geometric intersection between the treated area and the edges of the cell. We ran 1,000 simulations for each pair of treatment efficiency and radius values and derived the median and credible intervals from the generated samples.

### Data and code availability:

The code is publicly available in a [GitLab](https://gitlab.developers.cam.ac.uk/gilligan-epid/hlb) repository and the rasterised data are available via the University of Cambridge Data Repository [https://doi.org/10.17863/CAM.94186](https://eur03.safelinks.protection.outlook.com/?url=https%3A%2F%2Fdoi.org%2F10.17863%2FCAM.94186&data=05%7C01%7Ccag1%40cam.ac.uk%7C6ed75135d50f40fba2dc08db157dc822%7C49a50445bdfa4b79ade3547b4f3986e9%7C1%7C0%7C638127402184846429%7CUnknown%7CTWFpbGZsb3d8eyJWIjoiMC4wLjAwMDAiLCJQIjoiV2luMzIiLCJBTiI6Ik1haWwiLCJXVCI6Mn0%3D%7C3000%7C%7C%7C&sdata=LDX8OvZ2tuZW%2BHP4fLGQ%2FsTIVjt%2FXLnf%2FecfzNBIeVE%3D&reserved=0)

## Supplementary References

1. Gottwald T, Luo W, Posny D, Riley T, Louws F. A probabilistic census-travel model to predict introduction sites of exotic plant, animal and human pathogens. Phil Trans R Soc B. 2019;374: 20180260. doi:10.1098/rstb.2018.0260

2. Bayles BR, Thomas SM, Simmons GS, Grafton-Cardwell EE, Daugherty MP. Spatiotemporal dynamics of the Southern California Asian citrus psyllid (*Diaphorina citri*) invasion. Wang Z, editor. PLoS ONE. 2017;12: e0173226. doi:10.1371/journal.pone.0173226

3. Liu YH, Tsai JH. Effects of temperature on biology and life table parameters of the Asian citrus psyllid, *Diaphorina citri* Kuwayama (Homoptera: Psyllidae). Ann Applied Biology. 2000;137: 201–206. doi:10.1111/j.1744-7348.2000.tb00060.x

4. Parry M, Gibson GJ, Parnell S, Gottwald TR, Irey MS, Gast TC, et al. Bayesian inference for an emerging arboreal epidemic in the presence of control. Proc Natl Acad Sci USA. 2014;111: 6258–6262. doi:10.1073/pnas.1310997111

5. Gibson G, Renshaw E. Estimating parameters in stochastic compartmental models using Markov chain methods. Mathematical Medicine and Biology. 1998;15: 19–40. doi:10.1093/imammb/15.1.19

6. O’Neill PD, Roberts GO. Bayesian inference for partially observed stochastic epidemics. Journal of the Royal Statistical Society: Series A (Statistics in Society). 1999;162: 121–129. doi:10.1111/1467-985X.00125

7. Chib S, Greenberg E. Understanding the Metropolis-Hastings algorithm. The American Statistician. 1995;49: 327–335. doi:10.1080/00031305.1995.10476177

8. Gross D, Miller DR. The randomization technique as a modeling tool and solution procedure for transient Markov processes. Operations Research. 1984;32: 343–361. doi:10.1287/opre.32.2.343

9. Rabiner L, Juang B. An introduction to hidden Markov models. IEEE ASSP Mag. 1986;3: 4–16. doi:10.1109/MASSP.1986.1165342

10. Pooley CM, Bishop SC, Marion G. Using model-based proposals for fast parameter inference on discrete state space, continuous-time Markov processes. J R Soc Interface. 2015;12: 20150225. doi:10.1098/rsif.2015.0225

11. Dai Z, Wu F, Zheng Z, Yokomi R, Kumagai L, Cai W, et al. Prophage Diversity of ‘ *Candidatus* Liberibacter asiaticus’ Strains in California. Phytopathology. 2019;109: 551–559. doi:10.1094/PHYTO-06-18-0185-R

12. NOAA. NOAA National centers for environmental information, monthly national climate report for August 2016. U.S.A.: National Centres for Environmental Information, National Oceanic and Atmospheric Administration; 2016. Available: https://www.ncei.noaa.gov/access/monitoring/monthly-report/national/201608/supplemental/page-4
