# Supplementary figures and images for: Modelling the spread and mitigation of an emerging vector-borne pathogen: Citrus greening in the U.S
Source: PLoS Comput Biol. 2023 Jun 2;19(6):e1010156. doi: 10.1371/journal.pcbi.1010156 (PMC10266658; doi:10.1371/journal.pcbi.1010156)

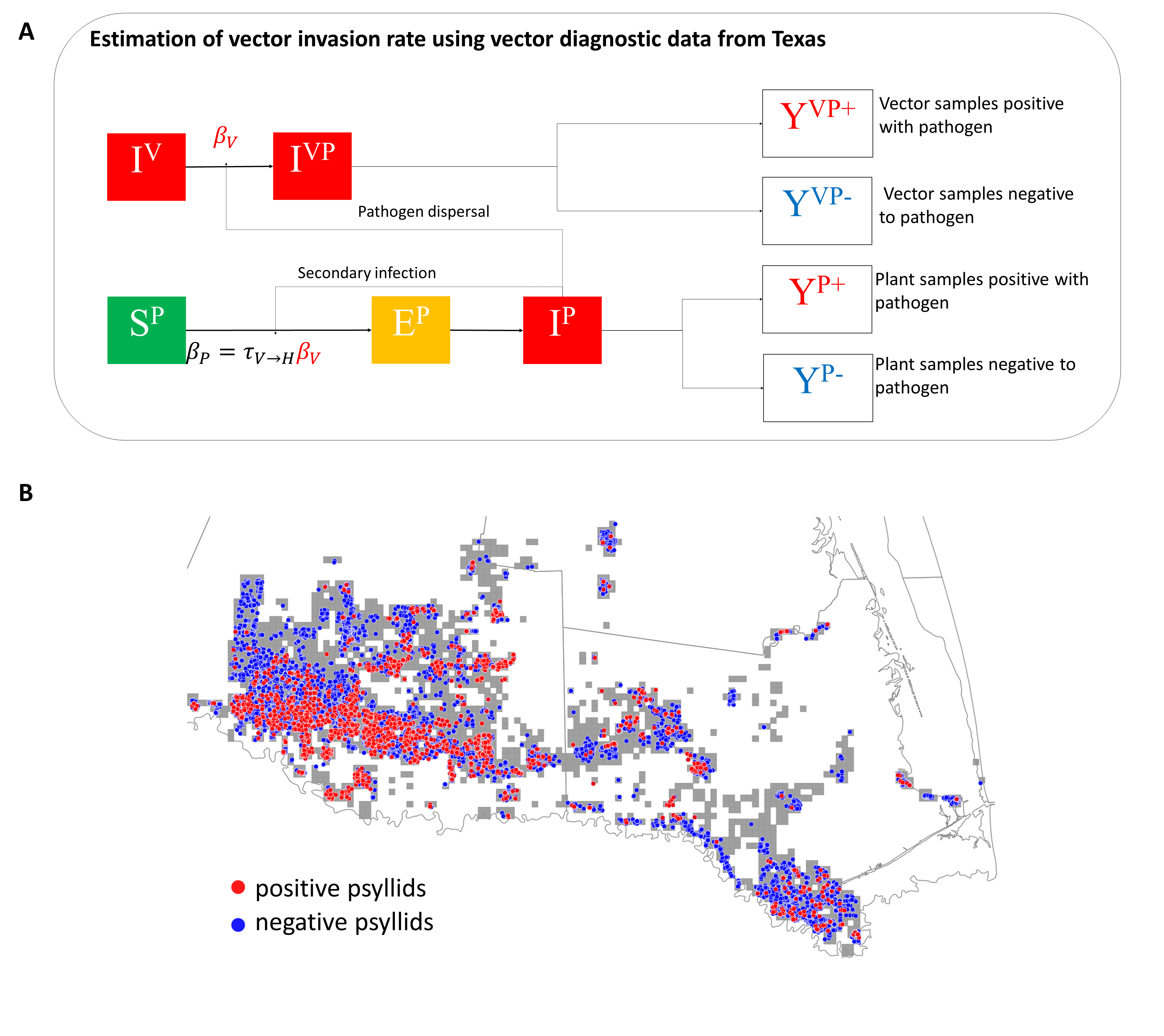

Supplement: S1 Fig — (A) The joint model of ACP and HLB spread after accounting for the fact that the vector had fully infested the citrus landscape before the modelling period. The ACP and HLB spread share the dispersal scale parameter and differ by the rates of invasion βV and transmission βP respectively. τV→H denotes the probability or efficacy of pathogen transmission to trees during the feeding of infected psyllids. The full model which allows for emerging ACP population is shown in Fig 2B (main text). (B) Geo-coded diagnostic samples of the vector ACP collected between December 2011 and October 2018 as part of the HLB state-wide survey in Texas. Samples with Ct value less than 38 were marked as positives. Diagnostic samples of citrus leaves collected as part of the same survey are mapped in Fig 1D (main text). (TIF) [file pcbi.1010156.s002.tif]

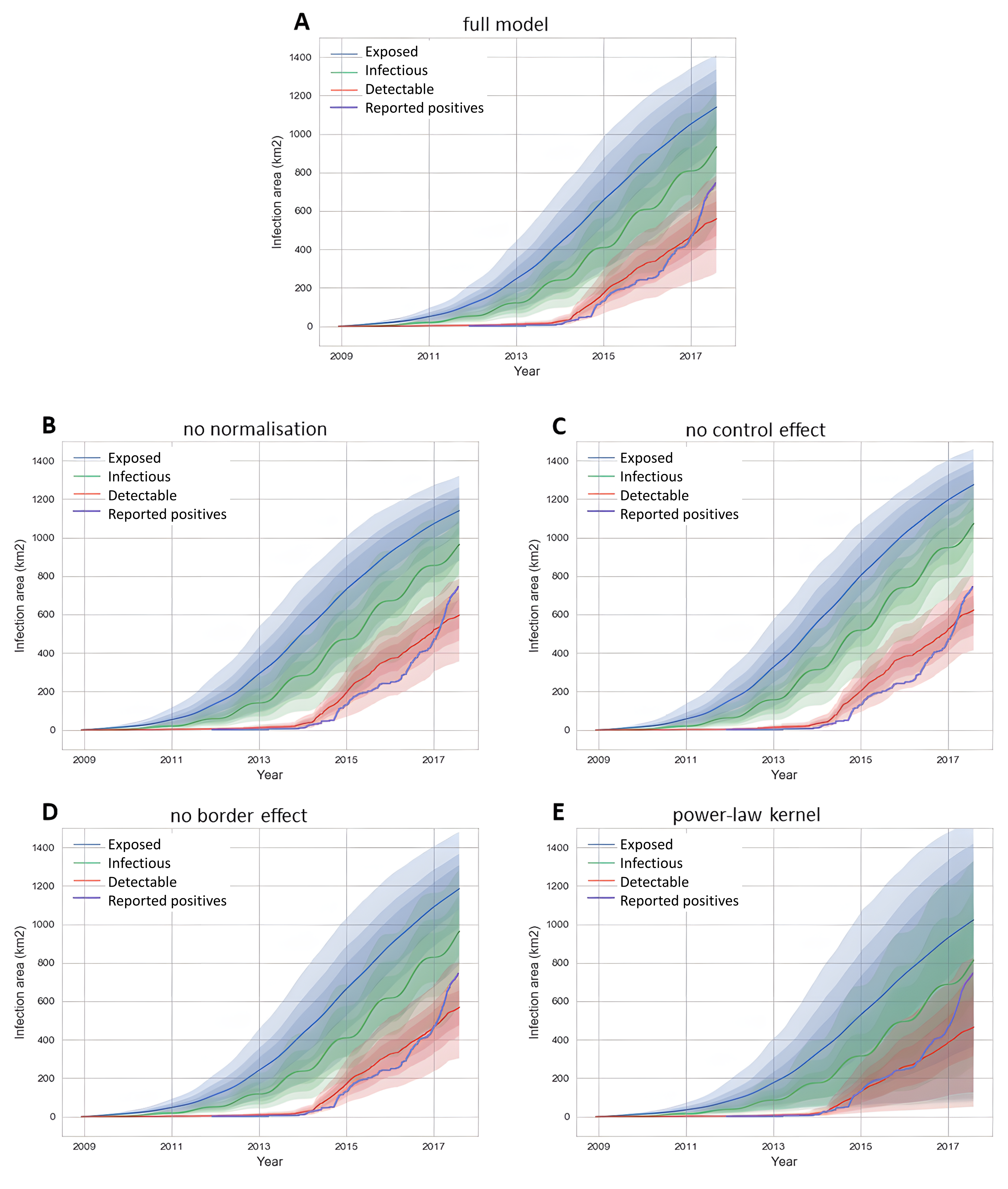

Supplement: S2 Fig — We considered the temporal progression of the prevalence of three infection categories (Exposed, Infectious, and Detected) for the HLB epidemiological model and its four model variants. Each model variant removes one key component from the HLB epidemiological model used throughout the paper. We show means of 500 simulation realizations as solid lines, and 50%, 75%, 95% credible intervals as shades of decreasing intensities. (A) The full HLB epidemiological model as described in the Methods section. (B) No normalisation, in which the normalisation term for vector fluxes is assumed to be the same for all cells and absorbed into the secondary infection rate. (C) No control effect, in which we ignored the occurrence of the annual coordinated spraying program. (D) No border effect, in which we did not distinguish between sites near to and far from the Mexico border and used the same primary infection rate for infected vector from external environments. (E) power-law kernel, in which the exponential dispersal function is replaced with a power-law function. (TIF) [file pcbi.1010156.s003.tif]

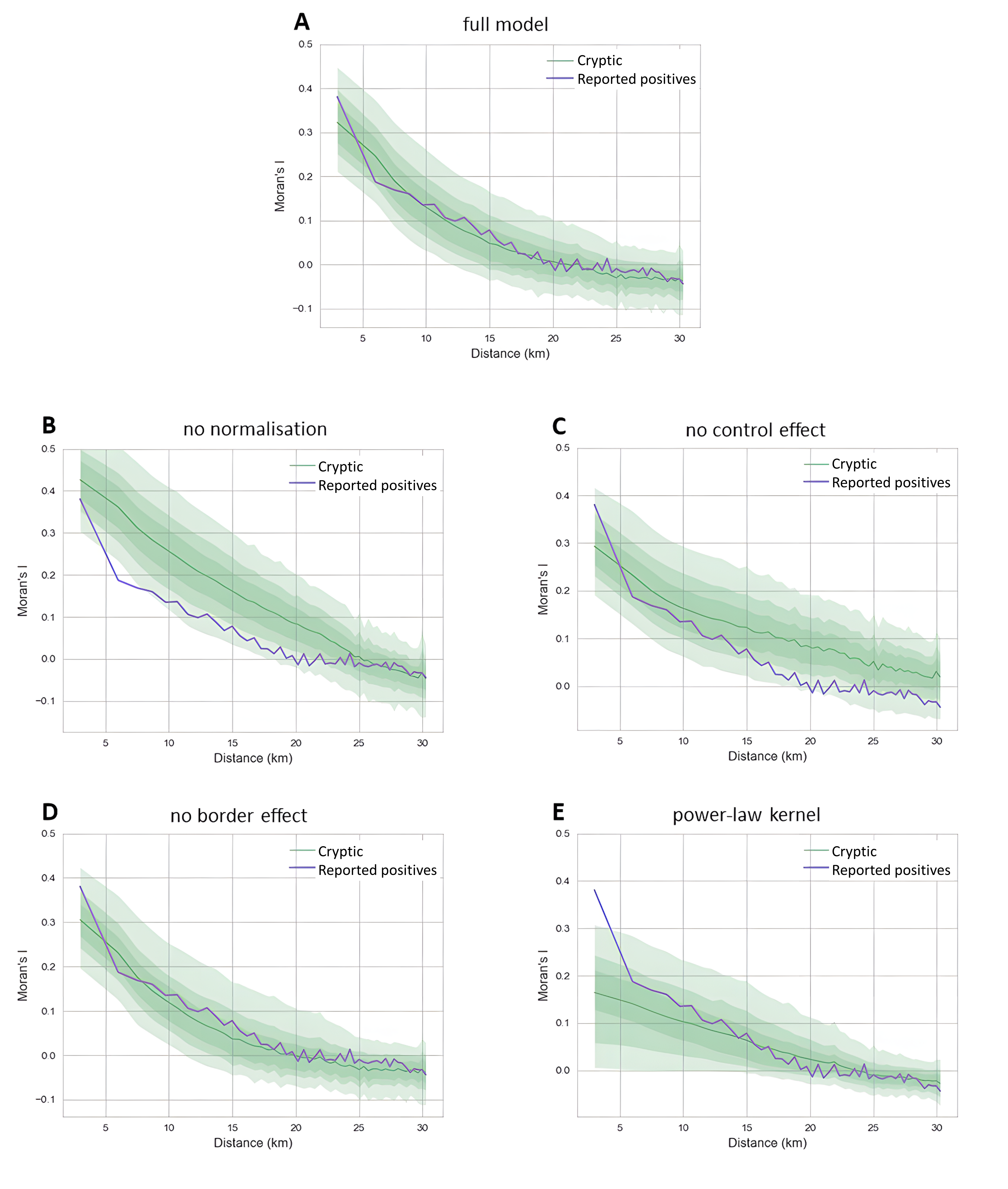

Supplement: S3 Fig — We considered the spatial autocorrelation scores of the Detected categories (green line and shades) with respect to the survey data (purple line) at the end of August 2017 for the HLB epidemiological model and its four model variants. (A) The full HLB epidemiological model as described in the Methods section. (B) No normalisation, in which the normalisation term for vector fluxes is assumed to be the same for all cells and absorbed to the secondary infection rate. (C) No control effect, in which we ignored the occurrence of the annual coordinated spraying program. (D) No border effect, in which we did not distinguish between sites near to and far from the Mexico border and used the same primary infection rate for infected vector from external environments. (E) power-law kernel, in which the exponential dispersal function is replaced with a power-law function. (TIF) [file pcbi.1010156.s004.tif]

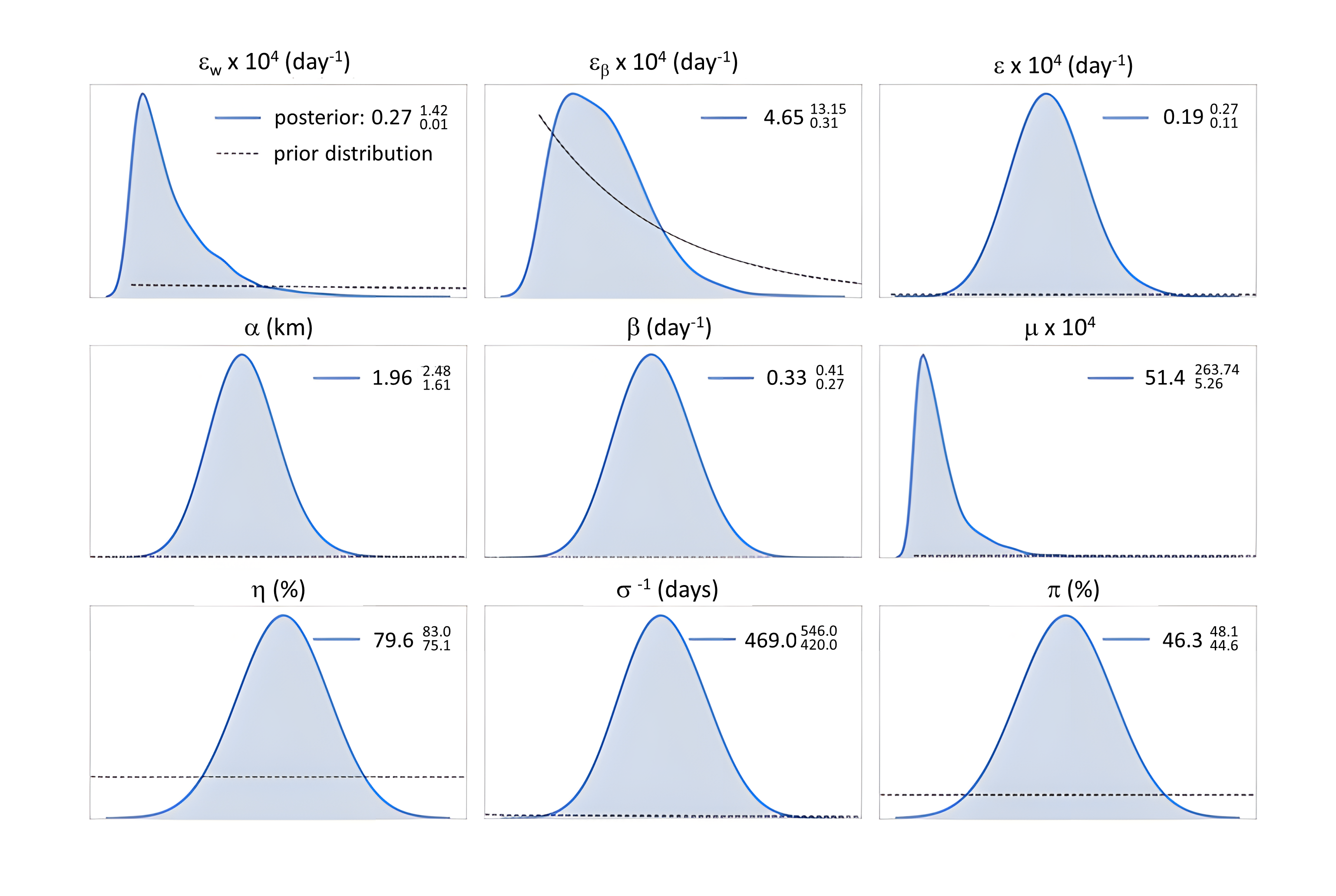

Supplement: S4 Fig — Posterior distributions of key parameters for HLB epidemic model in comparison with uninformative prior distributions (dotted black line). Legend numbers give the mean posterior value and the 95% credible interval for each parameter. (TIF) [file pcbi.1010156.s005.tif]
